# Gold Clusters on Graphene/Graphite—Structure and Energy Landscape

**DOI:** 10.1002/smsc.202400078

**Published:** 2024-08-12

**Authors:** Manoj Settem, Melisa M. Gianetti, Roberto Guerra, Nicola Manini, Riccardo Ferrando, Alberto Giacomello

**Affiliations:** ^1^ Dipartimento di Ingegneria Meccanica e Aerospaziale Sapienza Università di Roma via Eudossiana 18 00184 Roma Italy; ^2^ Institutt for maskinteknikk og produksjon NTNU Richard Birkelands vei 2B 7034 Trondheim Norway; ^3^ Dipartimento di Fisica Università degli Studi di Milano via Celoria 16 Milano 20133 Italy; ^4^ Dipartimento di Fisica dell’Università di Genova and CNR‐IMEM via Dodecaneso 33 16146 Genova Italy

**Keywords:** diffusion, gold, graphene, graphite, lubricity, nanoclusters

## Abstract

Adopting an advanced microscopic model of the Au–graphite interaction, a systematic study of Au nanoclusters (up to sizes of 11 238 atoms) on graphene and on graphite is carried out to explore their structure and energy landscape. Using parallel tempering molecular dynamics, structural distribution as a function of temperature is calculated in the entire temperature range. Low‐energy structures are identified through a combination of structural optimization and Wulff–Kaischew construction which are then used to explore the energy landscape. The potential energy surface (PES), which is energy as a function of translation and rotation, is calculated for a few Au nanoclusters along specific directions on carbon lattice. Minimum‐energy pathways are identified on the PES indicating a reduced barrier for pathways involving simultaneous rotation and translation. Diffusion simulations of Au_233_ on graphite show that diffusion mechanism is directly related to the PES, and the information of the cluster pinning events is already present in the PES. Finally, a comparison of various interaction models highlights the importance of reasonably correct Au–C interactions which is crucial for studying the energy landscape and cluster sliding.

## Graphical abstract

Structure and energetics of Au clusters on graphene and graphite are studied employing a state‐of‐the‐art Au–C interaction model. The calculated potential energy surface (PES), which is a function of translation and rotation, encodes detailed information about energy minima and barriers during diffusion. Diffusion of Au_233_ on graphite exhibits pinning events that precisely match the PES minima.

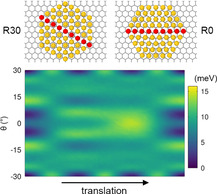

## 1 Introduction

The morphology of gold clusters has been widely investigated in recent years, in view of their relevance for catalysis,^[^ ^1^, ^2^, ^3^, ^4^, ^5^, ^6^, ^7^ ^]^ optical response,^[^ ^8^, ^9^ ^]^ and biocompatible applications.^[^ ^10^, ^11^, ^12^ ^]^ Most of these works focus on clusters formed by aggregation in the gas phase or in solution.^[^ ^13^, ^14^, ^15^, ^16^, ^17^ ^]^ However, a relatively common situation where gold clusters are relevant is when they aggregate after vapor deposition directly on solid surfaces.^[^ ^18^, ^19^, ^20^, ^21^ ^]^ Au clusters grown on graphite and graphene are examples of clusters grown on an especially flat and smooth surface.^[^ ^19^, ^20^, ^21^, ^22^, ^23^ ^]^

The Au—C interfaces are incommensurate so that their sliding can exhibit structural lubricity (or superlubricity) where the friction force is sublinear with the contact area.^[^ ^24^ ^]^ For these reasons, Au clusters on graphite or graphene are ideal candidates for studying friction. In this respect, it is known that size, shape, and orientation of Au clusters significantly influence the frictional behavior.^[^ ^21^, ^24^, ^25^ ^]^ Therefore, it is very important to determine the preferential shapes and orientations of these clusters.

On perfect graphene and graphite surfaces, Au atoms diffuse very quickly at room temperature and even below. When Au atoms meet, they can stick to each other and start the growth of clusters, whose shape is 3D but relatively flat.^[^ ^19^, ^20^, ^21^ ^]^ Nanosized clusters diffuse too on the perfect surfaces, with the result that, experimentally, clusters are never observed at the middle of flat terraces, but rather at surface defects where they can get trapped.^[^ ^20^, ^26^ ^]^

Au adatoms and clusters on graphene/graphite have been studied extensively using empirical force fields and density functional theory (DFT). According to DFT calculations,^[^ ^27^ ^]^ Au adatoms prefer the atop site (Au atom positioned above a C atom) followed by the bridge site (Au atom positioned above the midpoint of C—C bond) and the hollow site (Au atom positioned above the center of C hexagon). Depending on the type of pseudopotential, the energy ordering of the bridge and hollow sites can reverse,^[^ ^28^ ^]^ with the atop site being the best in all cases. The preference of Au adatoms for atop sites has been verified experimentally^[^ ^29^ ^]^ as well.

Due to the size limitations inherent to DFT calculations, the energetics and dynamical behavior of larger Au clusters are usually studied using empirical force fields.^[^ ^30^, ^31^ ^]^ In these studies, the Au—C interaction is modeled with Lennard–Jones (LJ) potential which predicts the hollow site to be most favorable adsorption site followed by bridge and atop sites (**Table** **1**) in disagreement with both experimental and DFT studies.

**Table 1:** Adsorption energy ($E_{\text{ads}}$) of Au adatom at atop, bridge, and hollow sites with LJ and SAIP potentials for Au–C interaction. The LJ Au–C parameters are taken from ref. [54]. The adsorption energies are reported with respect to the adsorption energy of the atop site. For reference, the values according to DFT^[^ ^27^ ^]^ are also reported.

	$E_{\text{ads}}$ [meV]		
	LJ	SAIP	DFT
Atop	0	0	0
Bridge	–4.45	0.27	5
Hollow	–31.44	1.23	22

In contrast, a recently developed semi‐anisotropic interfacial potential (SAIP)^[^ ^32^ ^]^ predicts accurately the energy ordering of the adsorption sites placing atop as the best site (Table 1), although the energy differences are lower compared to DFT.^[^ ^27^, ^28^ ^]^ A critical component of the Au—C interfaces is their mutual orientation (or “twist” angle) which influences the energy landscape^[^ ^33^ ^]^ and also the frictional properties.^[^ ^21^ ^]^ The unphysical prediction of the best absorption site is likely to affect LJ capability to predict preferred orientations. We therefore adopt a SAIP model for the Au—C interaction and apply it to study the optimal structure, location, and orientation of Au clusters on graphene. Additionally, we analyze the currently unavailable structural distribution (relative abundance of different structure kinds as a function of temperature) of Au clusters supported on graphene.

Specifically, we perform a systematic computational analysis of the structures and energetics of gold nanoclusters supported on graphene or on multiple layers of graphite. First of all, we identify the optimal shapes, adsorption sites, and orientations with respect to the substrate for a set of cluster sizes in the range up to 147 atoms. These optimal structures are determined by global optimization searches with basin hopping (BH).^[^ ^34^, ^35^ ^]^ For Au_147_ and Au_55_, we perform a full temperature‐dependent analysis of the equilibrium structures by parallel tempering molecular dynamics (PTMD).^[^ ^36^ ^]^ The structures of supported clusters are compared also to those of free, gas‐phase ones in order to asses the influence of the substrate. Over a broader range of cluster sizes, we identify the optimal structures by means of the Wulff–Kaischew (WK) construction.^[^ ^37^, ^38^ ^]^ Finally, we explore the energetics of these clusters as a function of the location and angular orientation on the substrate in order to determine the local minima and the barriers against sliding. These results are informative not just regarding the cluster equilibrium and statistical properties but also their dynamical behavior in nanomanipulation experiments.^[^ ^19^, ^21^, ^39^ ^]^

## 2 Results and Discussion

**Figure 1:**
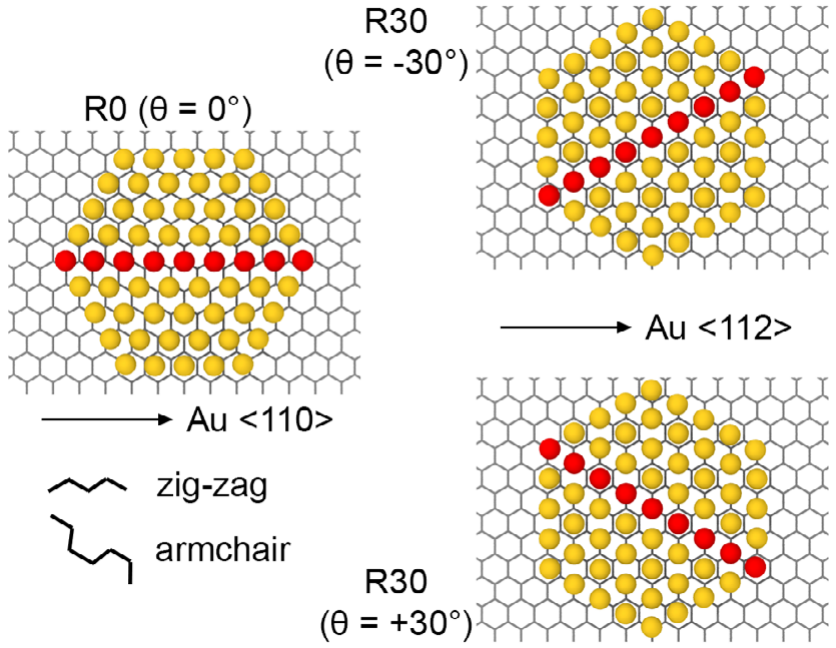
Conventions for the orientation of a Au cluster supported on graphene/graphite. A single carbon and just the contact gold layer are depicted. θ=0° (named R0) indicates the alignment where a Au ⟨110⟩ direction is parallel to a zigzag direction of graphene. Positive *θ* implies clockwise rotation. A θ=±30° rotation leads to a R30 orientation, with the Au ⟨110⟩ direction aligned parallel to armchair directions of graphene, and the Au ⟨112⟩ direction parallel to the zigzag of graphene.

We use molecular dynamics (MD) simulations and structural optimization to study the ground and low‐energy configurations of Au clusters adsorbed on monolayer graphene or on the surface of bulk graphite. Studying the structure of Au clusters on graphene/graphite is particularly relevant to contrast with other synthetic routes: in gas phase vs. at the surface. While the structure of large Au clusters on graphitic substrates^[^ ^23^ ^]^ is well known, to the best of our knowledge, a detailed investigation of structural populations of small clusters was not previously carried out. Details about the atomic configuration and interactions are provided in Section 4. Au exhibits a strong tendency to form fcc/hcp nanoparticles that contact the graphene layer through a triangular lattice. Due to the extreme flatness of graphene and the relatively weakly corrugated Au—C interaction, several mutual translation and orientation arrangements compete. To identify the orientation of Au clusters relative to the graphene/graphite lattice, we use the convention illustrated in **Figure** **1**. The reference θ=0 angle, called “R0 orientation” in the literature, has the Au ⟨110⟩ directions parallel to the zigzag directions of graphene. Clockwise rotations lead to positive *θ*. A rotation of ±30° results in a “R30 orientation,” in which the zigzag directions of C lattice are now parallel to the Au ⟨112⟩ directions.

### 2.1 Structure of Supported Au Clusters

As a reference, we consider Au_147_ to understand the differences between the structure of gas‐phase (unsupported) and supported Au clusters. The structural distribution (fraction of various structural classes as a function of temperature) of unsupported Au_147_ was reported previously.^[^ ^40^, ^41^ ^]^ In the gas phase, the global minimum exhibits the symmetry of a decahedron (Dh). This is also the dominant motif up to higher temperatures before melting. Locally stable icosahedral (Ih) structures are observed above 400 K and become significant close to melting. Small amounts of face‐centered cubic (fcc), mix‐fcc‐hcp (fcc with faults such as twins, stacking defects, or even entirely hexagonal close‐packed) locally stable structures are observed at all temperatures.

**Figure 2:**
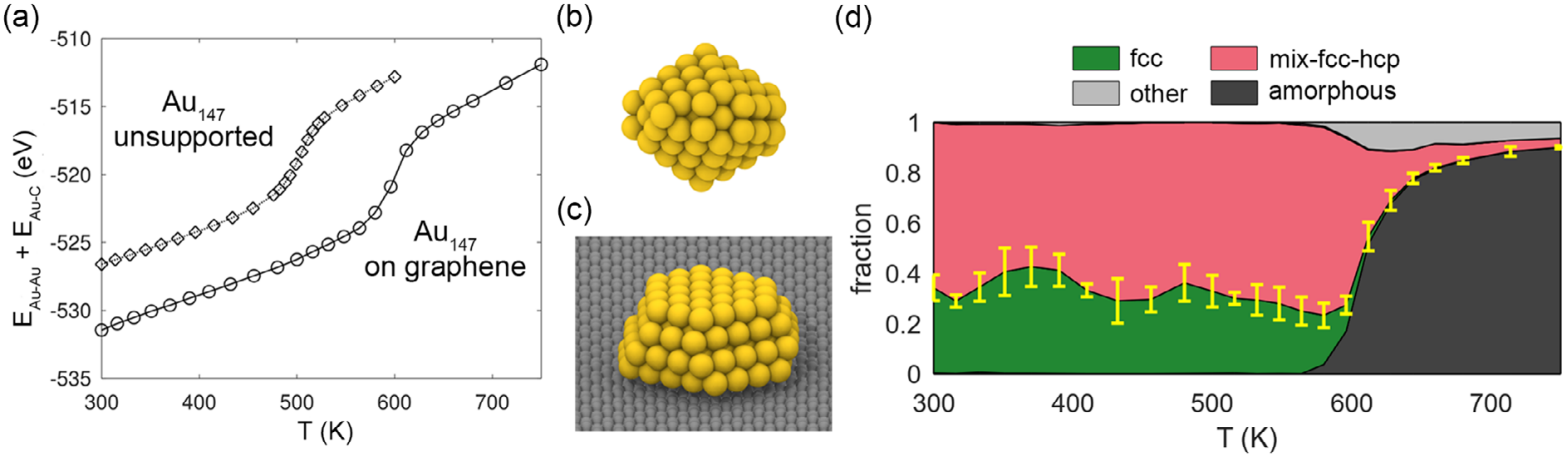
a) Comparison of the time‐averaged total potential energy (caloric curves) of Au_147_ unsupported and deposited on graphene. The rounded steps are indicative of the melting transition. The global potential energy minimum of Au_147_ b) unsupported and c) on graphene. d) Distribution of graphene‐adsorbed Au_147_ structure types. The error bars represent the cumulative error of fcc and mix‐fcc‐hcp fractions.

On graphene, Au clusters exhibit morphologies that differ significantly from unsupported ones. To begin with, we find an increased thermal stability (**Figure** **2**a) with Au_147_ showing a raise in the melting point (the step in the caloric curve) from ≈505 K (unsupported) to ≈596 K (on graphene). With regard to the structure, Au clusters on graphene form 3D but relatively flatter geometries as compared to unsupported Au clusters. Figure 2b shows the global Dh minimum of unsupported Au_147_. By comparison, Figure 2c displays the best structure on graphene, namely a 4‐layer, hexagonal close‐packed (hcp) arrangement. Figure 2d reports the distribution of different (meta)stable structure kinds of Au_147_ on graphene. We find four main structural classes: fcc, mix‐fcc‐hcp, other, and amorphous, along with statistically negligible amounts of decahedra and icosahedra. Interestingly, noncrystalline structures (decahedra and icosahedra) are however observed to grow on amorphous carbon substrates.^[^ ^42^ ^]^ Right up to melting, almost all the structures are either fcc or mix‐fcc‐hcp. Close to melting, we observe an increasing amount of other structures which are typically combinations of crystalline and amorphous regions within the same cluster. The fraction of fcc appears to fluctuate as a function of *T*. Such fluctuations would most likely decrease if one could afford a longer PTMD simulation time than our 75 ns. The cumulative error in the fraction of fcc and mix‐fcc‐hcp (see Section 4) is also displayed in the fraction plot (in yellow). The “wavy” fluctuation is within the error bar.

A key difference with the distribution of structure classes of unsupported Au_147_ is the lack of decahedra and icosahedra at all temperatures. This is mainly due to the hexagonal arrangement of the underlying graphene substrate guiding the cluster formation: clusters consisting of stacked close‐packed layers, either as fcc or mix‐fcc‐hcp structures, exhibit optimal matching with the graphene substrate. This is illustrated by comparing mix‐fcc‐hcp (global minimum which is completely hcp) with a decahedral cluster (Figure S1 in the Supporting Information). In the hcp cluster (Figure S1a,c, Supporting Information), the Au—C interface is relatively flat with pseudo‐commensuration at the R30 orientation (the periodic unit is marked in Figure S1c, Supporting Information). This is not the case with the decahedral cluster (Figure S1b,d, Supporting Information). The decahedral axis is roughly parallel to the Au—C interface. In order to maintain a reasonably flat interface, the two subunits in the lower left are distorted resulting in an energetically poor arrangement of the Au interfacial layer (see bottom view in Figure S1d, Supporting Information). Geometric constraints of this kind explain the dominance of fcc or mix‐fcc‐hcp structures at all temperatures (Figure 2d).

**Figure 3:**
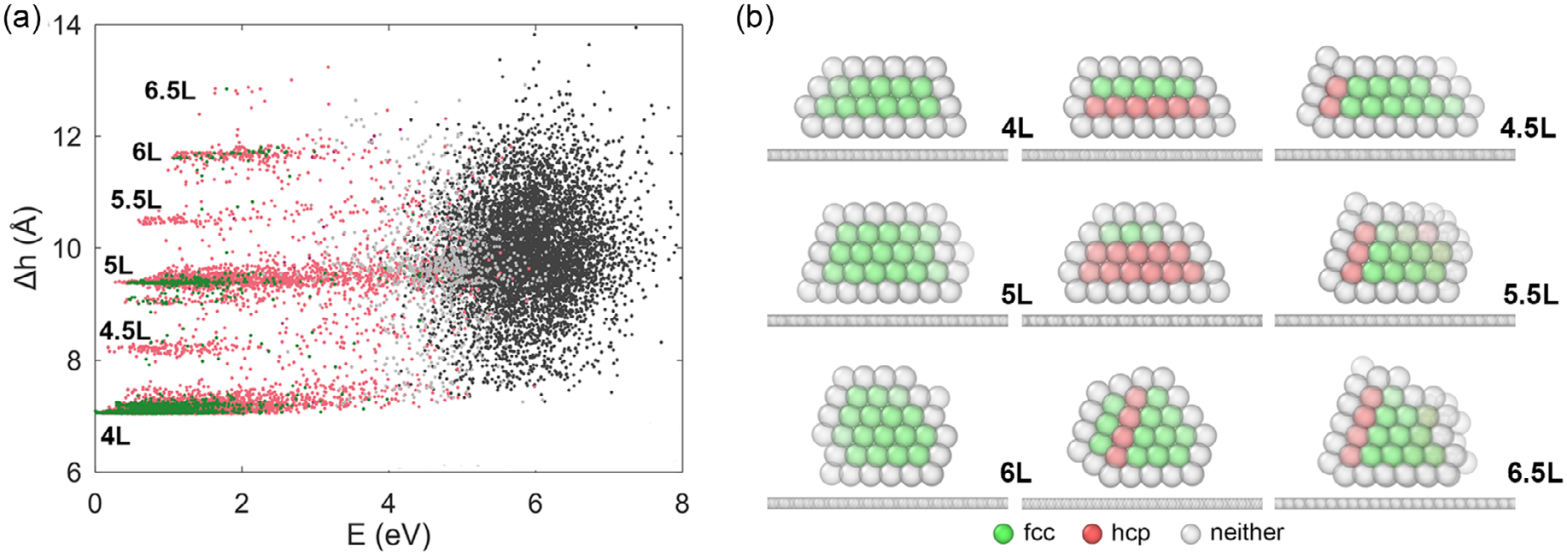
a) The Au_147_ cluster thickness Δh, correlated with the local‐minimum excess potential energy. A point's color indicates the structure kind, with the same notation as in Figure 2d. b) Side view of a few representative structures consisting of four (4L), five (5L), or six (6L) Au layers. M.5L stands for structures exhibiting intermediate thicknesses. The left column reports fcc structures. The center and right columns report mix‐fcc‐hcp structures. Ball colors mark the local atomic coordination, as indicated in the legend.

Another key geometrical feature is the thickness of Au clusters, which is dependent on the wettability of Au on graphene. Given that the graphene substrate is parallel to the xy plane, here thickness refers to the *z* coordinate. We define the cluster thickness as the difference $\Delta h=z_{\text{top}}-z_{\text{bot}}$, where $z_{\text{top}}$ and $z_{\text{bot}}$ are, respectively, the *z* coordinates of the topmost and bottommost atom within the cluster. **Figure** **3**a reports the thicknesses of all the Au_147_ locally stable structures sampled from PTMD (28 800 configurations) as a function of their potential energy (after local relaxation). Energy is measured relative to the global minimum. The layered arrangement of close‐packed planes (either as fcc or mix‐fcc‐hcp) in Au clusters is evident from the “discretized” thickness of the analyzed local minima. Structures up to an excess energy of ≈4 eV are neatly arranged into bands containing structures with four layers (4L), five layers (5L), and six layers (6L). Representative 4L, 5L, and 6L structures are displayed in Figure 3b. In addition, we observe bands of structures with intermediate thicknesses. Consider the structures denoted as 4.5L: these structures typically are mix‐hcp‐fcc characterized by a stacking defect that is not parallel to the Au—C interface; see Figure 3b for examples. As a result, certain atoms within the cluster occupy *z* positions intermediate between the fourth and fifth layers.

To confirm the trends observed for Au_147_, we undertake a similar analysis for Au_55_. Similar to Au_147_, fcc and mix‐fcc‐hcp dominate up to melting (Figure S2a in the Supporting Information). However, this smaller cluster exhibits a larger proportion of *other* structures, especially close to melting. Apart from partial disorder, *other* structures of Au_55_ are associated with local rearrangements close to the Au—C interface which results in icosahedral features. Such local rearrangements have been observed in unsupported Au clusters too.^[^ ^40^, ^43^ ^]^ Studying Au_55_, we also observe the discretized cluster thicknesses that indicate a layered arrangement.

The results of Au_147_ and Au_55_ emphasize that Au clusters grown on graphene exhibit an overwhelming tendency to form shapes that are the result of stacking several close‐packed triangular layers. However, this observation does not provide complete information regarding the shape of Au clusters.

Carrying out PTMD simulations for larger clusters is computationally expensive. As a way around, we start from the bulk fcc structure of gold and apply the WK construction^[^ ^37^, ^38^ ^]^ which provides approximate indications about the optimal shape of a supported particle. The global minimum structures of small Au clusters have only (111) facets in contact with the C lattice. Hence, for energetics and diffusion studies, we considered only those WK clusters that satisfy this geometrical property. A few of the WK shapes are then confirmed to be global minima using BH searches.

**Figure 4:**
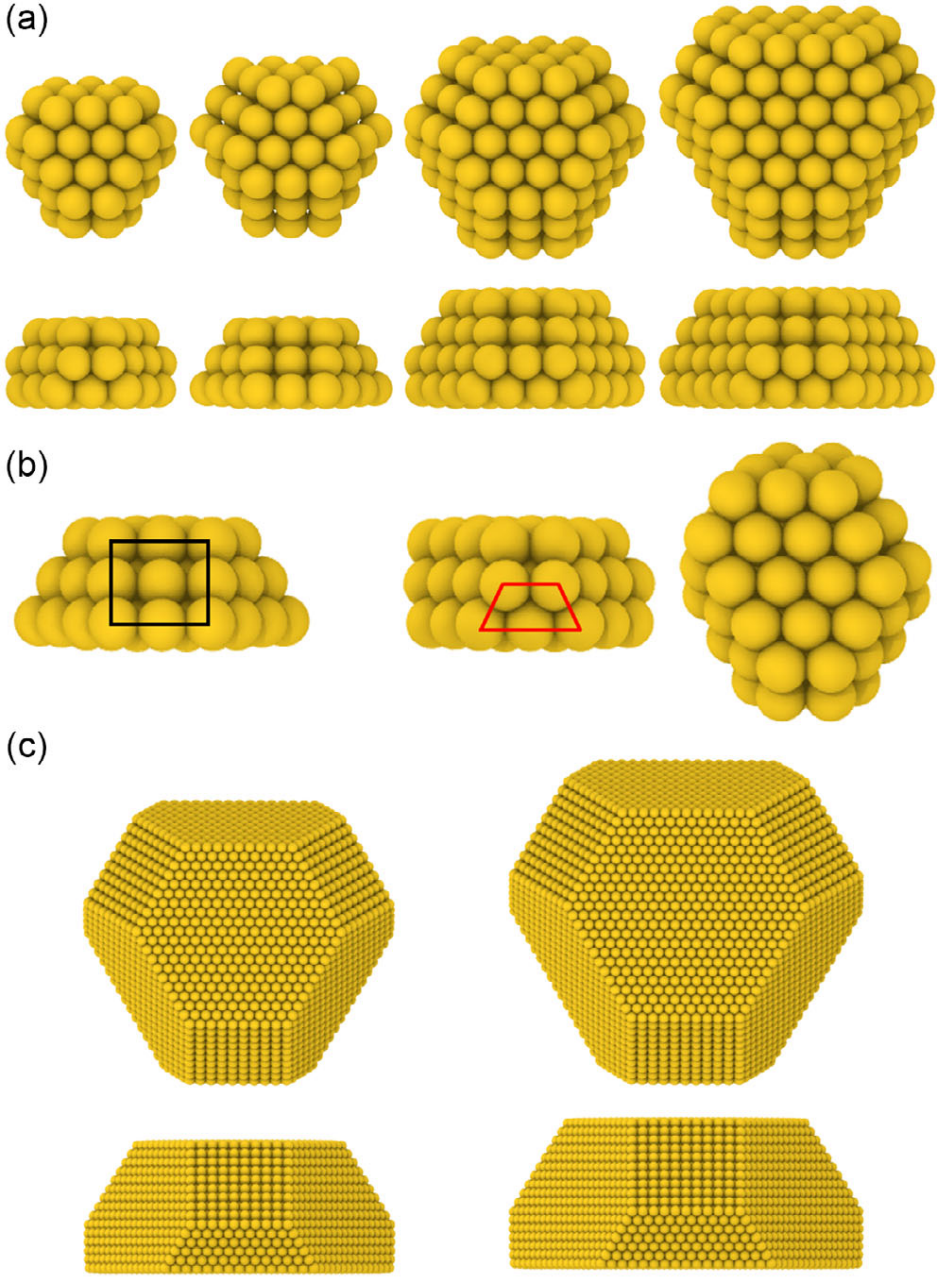
a) Top and side view of WK Au clusters on graphene (left to right): Au_49_, Au_58_, Au_119_, and Au_157_. b) Comparison of two Au_58_ structures. Left: structure obtained from the WK construction, side view; center: side view, and right: top view of the energy global minimum. A (100) side facet in the WK structure is marked in black and a (111) side facet in the global minimum is marked in red. c) Top and side view of (left) Au_7595_ and (right) Au_11 238_ WK structures.

**Figure** **4**a shows WK structures of graphene‐deposited Au clusters consisting of 49, 58, 119, and 157 atoms. For these sizes, we also carried out BH searches to identify the global minimum. With the exception of Au_58_, the WK structures are the global minima. The discrepancy in the case of Au_58_ can be understood by comparing the geometry of the WK and the global minimum structures (Figure 4b) with respect to the side facets in contact with graphene. The following feature is common to all the global minima: only (111) facets meet the graphene substrate. The WK Au_58_ structure violates this rule, with even (100) facets meeting graphene. Under such scenarios, asymmetric cluster shapes are energetically preferred such as the one shown here for Au_58_ and those previously described for Au_55_ and Au_147_. Hence, for studying the energy landscape of Au clusters on graphene/graphite, we focus on WK clusters with only (111) facets in contact with graphene/graphite. Figure 4c displays two examples of larger WK Au structures (Au_7595_ and Au_11 238_), precisely with this property of the facets in contact with the substrate. Shapes resembling the WK Au clusters have been observed in dewetted Au particles on graphene.^[^ ^23^ ^]^ The fraction of such shapes is found to increase with thermal annealing temperature suggesting that WK shapes are indeed equilibrium shapes.

### 2.2 Energetics of Au Clusters on Graphene/Graphite

**Figure 5:**
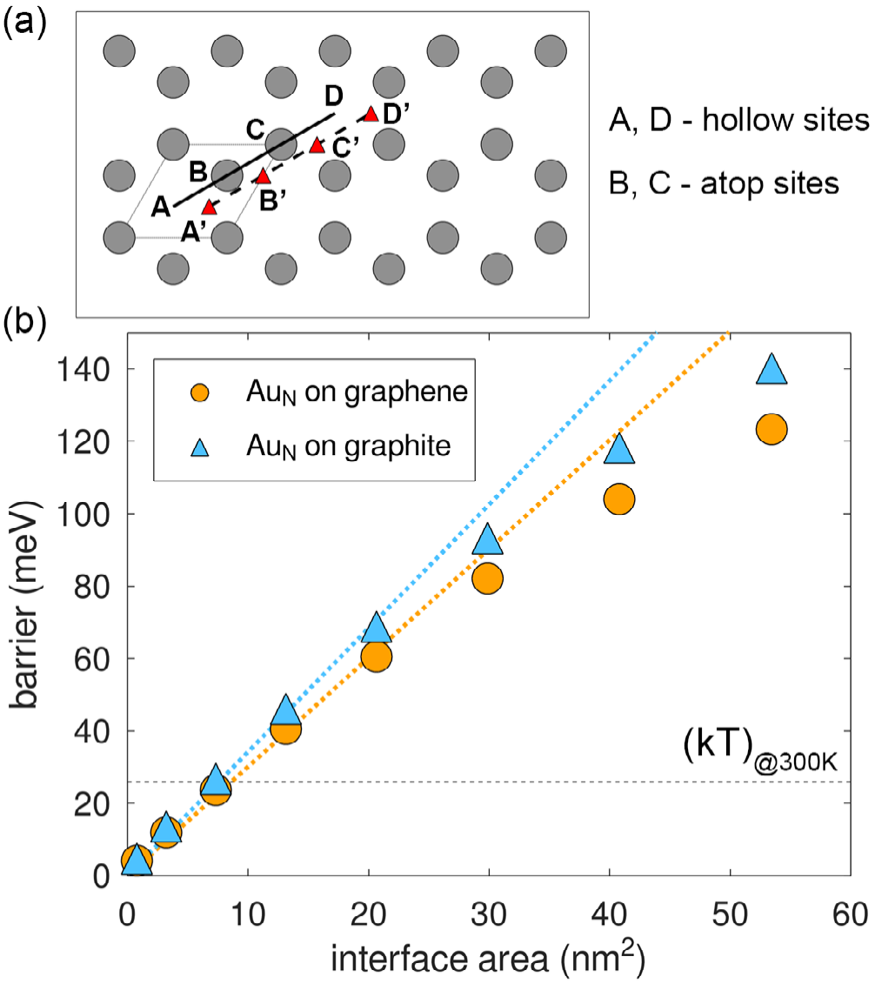
a) Graphene lattice indicating the unit cell and the positions of *hollow* (A, D) and *atop* (B, C) sites The sites A', B', C', and D' are generated by shifting the sites A, B, C, and D by 1/3 of the *x*‐directed unit cell vector. b) Contact‐area dependence of the pure translational energy barriers of WK R30‐oriented Au_*N*_ clusters (49≤N≤11 238) with a hexagon‐shaped layer in contact with the graphene (circles) and graphite (triangles) lattices. The dotted lines are linear fits passing through the origin of the five smallest‐size points, up to ≈22 nm^2^. The horizontal black dashed line marks the 300 K thermal energy.

We now examine the energetics of Au clusters on graphene/graphite, a property which is not directly accessible from experiments. We calculate the energy barrier that contrasts a pure translation of the center of mass (COM) (in the xy plane) of the Au cluster in contact with the C lattice from a *hollow* site (A) to an adjacent *atop* site (B), as illustrated in **Figure** **5**a. Figure 5b reports the energy barriers for WK clusters (size: 49 to 11 238 atoms) having a regular hexagon‐shaped contact layer in the R30 orientation on graphene and graphite. First, the barriers are quite small, less than 150 meV for all considered clusters. The energy barriers for the first three reported clusters (Au_49_, Au_233_, and Au_708_) are even smaller than the room‐temperature thermal energy. Second, the energy barrier increases roughly linearly with the interface area up to ≈20 nm^2^ and then deviates from linearity for larger WK clusters. As a result, we should expect small Au clusters to diffuse rapidly on graphene/graphite. This is in line with experiments on small Au clusters, which are seldom observed on pristine graphene/graphite surfaces but are typically found pinned at defect sites.^[^ ^20^ ^]^

**Figure 6:**
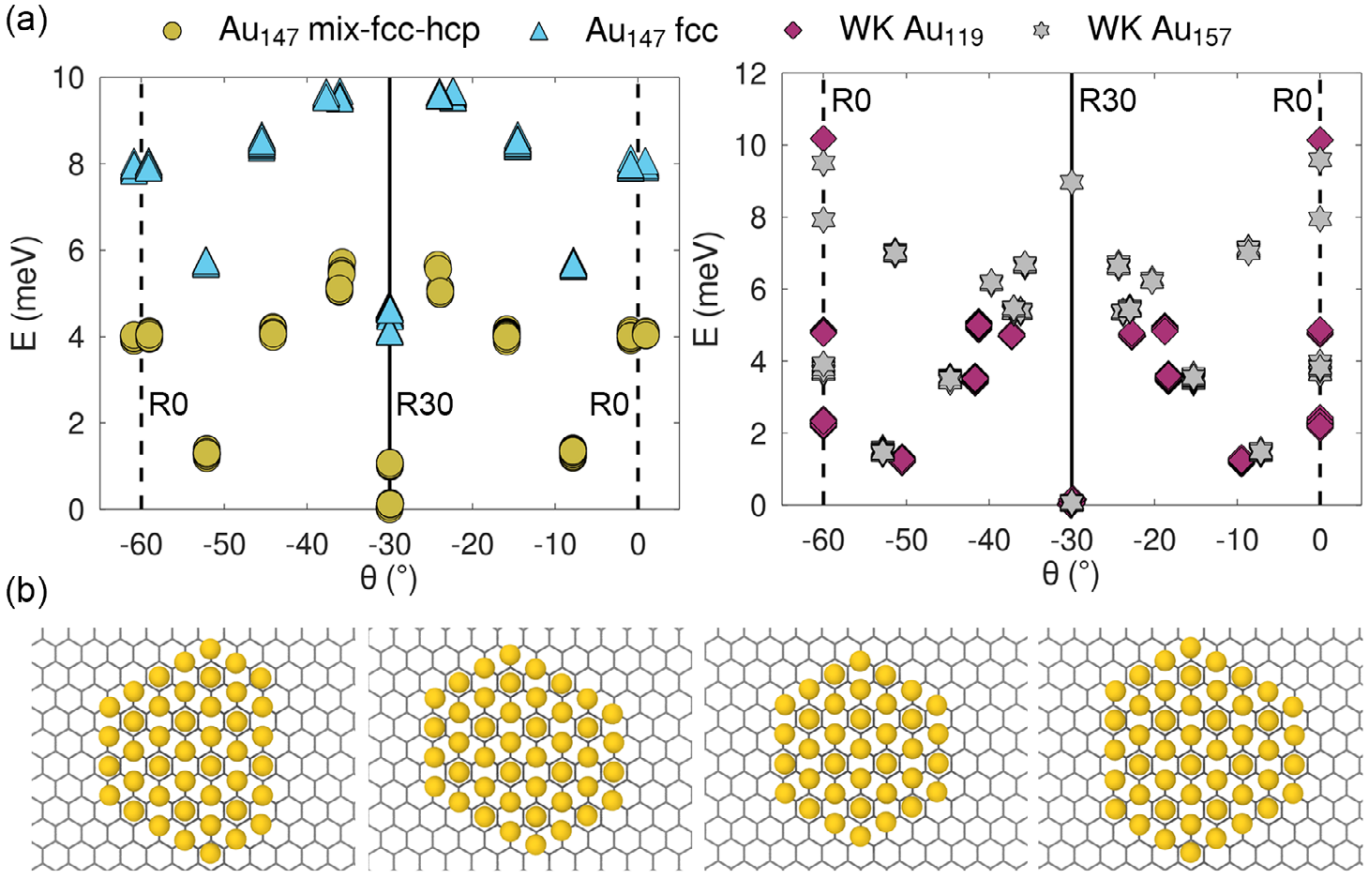
a) Excess energy of Au clusters on graphene as a function of their orientation *θ*. b) Au layer in contact with graphene in the lowest‐energy R30 configuration: (from left to right) Au_147_ mix‐fcc‐hcp, Au_147_ fcc, WK Au_119_, and WK Au_157_.

The energy of Au clusters on graphene/graphite depends also on their orientation. For studying the orientation effects, we consider the two lowest‐energy configuration of Au_147_ belonging to mix‐fcc‐hcp and fcc motifs obtained from PTMD. Along with these structures, we also consider WK Au_119_ and WK Au_157_ clusters. **Figure** **6**a reports the *excess* energy of these four examples of Au clusters as a function of their orientation. This is obtained by placing the COM of the Au cluster at the sites A, B, C, A′, B′, and C′ of a rigid graphene substrate (see Figure 5a) and scanning the initial rotation angle from 0° to 60° in steps of 1° while allowing the Au atoms to relax fully. When the Au contact layer has threefold rotational symmetry, the R30 orientations provide stable local minima only at the sites A′, B′, and C′, and hence these sites are also considered (see Section S3 in the Supporting Information for further details). Depending on the initial angle, the fully relaxed clusters fall into a nearby local minimum, characterized by a specific orientation and location on the graphene lattice. After the clusters relax, we evaluate their final orientation angle (see Section S4 in the Supporting Information for details on how the orientation angles are measured). Figure 6a reveals that the orientation of the local minima depends on the shape of the Au layer in contact with graphene.

We denote the shape of a Au contact layer by the length (number of atoms) of its edges beginning from its shortest edge and moving clockwise. For these clusters, the Au contact layers have a hexagonal shape with the following edge lengths (see Figure 6b): Au_147_ mix‐fcc‐hcp (3‐6‐3‐5‐4‐5), Au_147_ fcc (4‐4‐5‐4‐4‐5), WK Au_119_ (4‐4‐4‐4‐4‐4), and WK Au_157_ (4‐5‐4‐5‐4‐5). Similar shapes have been observed for larger Au particles (≈50 nm) grown on graphene.^[^ ^23^ ^]^ **Table** **2** reports the orientations of the three lowest‐energy minima of each Au cluster. R30 is the orientation of the global minimum for all these clusters. In Au_147_, R30 is also the second lowest‐energy minimum with a third minimum occurring near −8°. For the WK clusters, the second and occasionally the third lowest‐energy minimum occur at nontrivial angles; R0 local minima are present too, occasionally higher up in energy. In Au_147_ clusters, the fourth minimum sits near R0, at θ=±1°. Similar angular deviations away from R0 have been observed experimentally for Au clusters (having similar size and shape to the ones considered here) pinned at defects sites.^[^ ^20^ ^]^ Interestingly, for the clusters studied here, we do not observe any Novaco–McTague^[^ ^44^ ^]^ alignment, and in fact all clusters favor the R30 orientation as the global minimum. This holds even for larger sizes, Au_6710_ and Au_7595_, as discussed in the following section. This lack of a Novaco–McTague alignment is expected for two different reasons: for small‐size contacts, effective pseudo‐commensuration takes places favoring trivial (R30) alignment; for clusters of sizes few times the moiré pattern spacing, the edge, especially when consisting of straight segments,^[^ ^45^ ^]^ favors trivial alignment too, as also observed experimentally.^[^ ^24^ ^]^ Second‐ or third‐best minima associated with nontrivial angles near R0 may be affected by Novaco–McTague physics. Finally, we note that our scan based on a relatively sparse array of initial cluster positions may have missed a few local minima.

**Table 2:** Orientation of the first three lowest‐energy configurations of Au clusters on graphene, corresponding to Figure 6a.

Energy rank	Au_147_ mix‐fcc‐hcp	Au_147_ fcc	WK Au_119_	WK Au_157_
Lowest	R30	R30	R30	R30
Second	R30	R30	±9.5°	±7.2°
Third	±7.9°	±7.8°	R0	±15.3°

#### 2.2.1 Potential Energy Surface (PES)

As anticipated, the energy of an Au cluster depends on its orientation, along with the location of its COM. Hence, we calculate the roto‐translational potential energy surface (PES) in order to gain insights into the energy landscape of Au clusters on graphene/graphite. We adopt the following procedure. We choose a specific direction along the carbon lattice and discretize it into grid points. Each grid point corresponds to a specific value of the fractional distance along this direction with values 0 and 1 indicating the end points. We position the COM of the Au cluster (in its global minimum obtained from a full relaxation) at each grid point and then scan the rotation range of −30° to +30° in steps of 1°. At each combination of fractional distance and rotation angle (*θ*), we measure the energy of the Au cluster on rigid graphene. In order to lock the orientation and position of the Au cluster, we allow only the *z* coordinate of Au atoms to relax, keeping x,y fixed.

**Figure 7:**
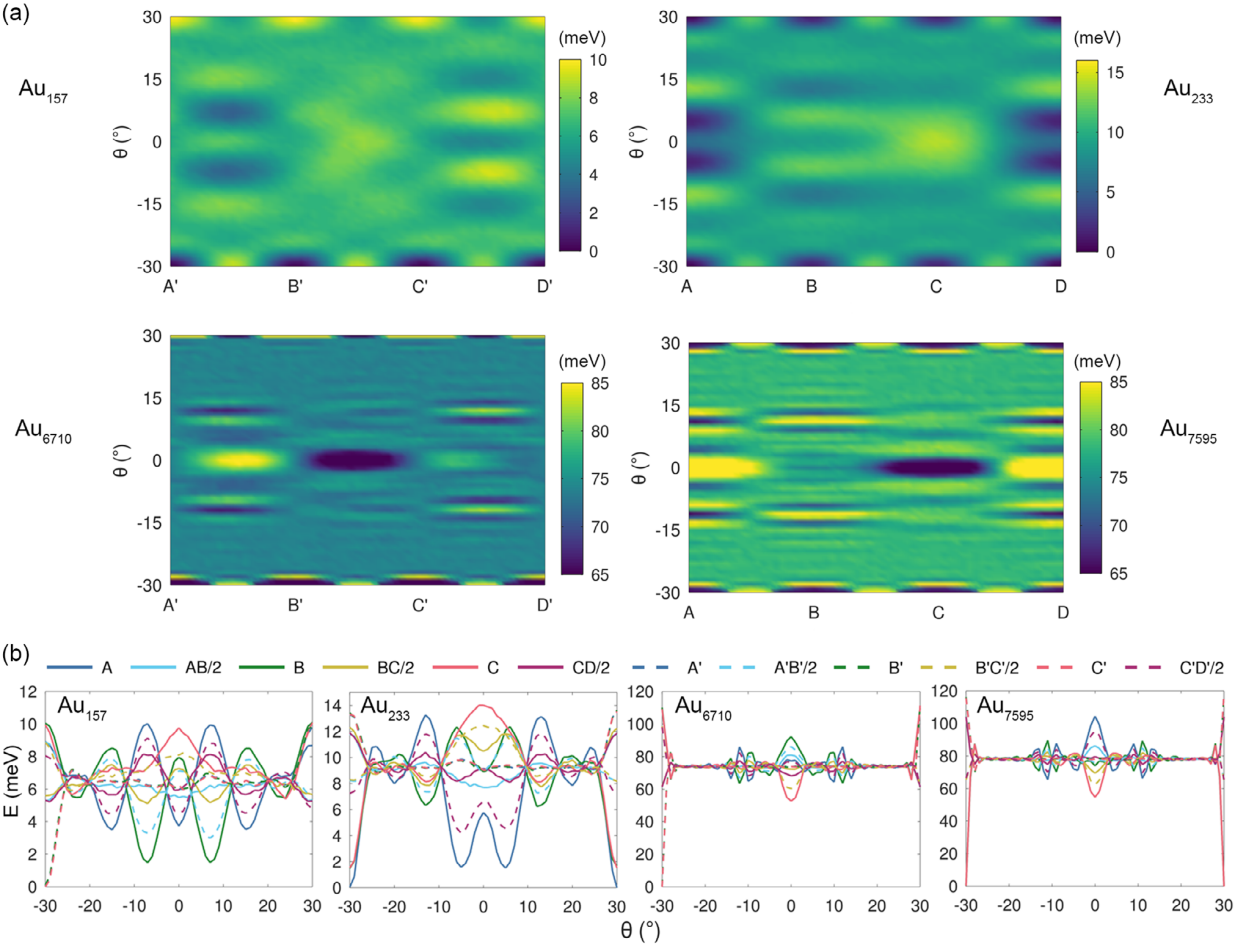
a) Roto‐translational energy surface (PES) of WK clusters on graphene along the lines A'D' for Au_157_ and Au_6710_, and along AD for Au_233_, Au_7595_. These paths are shown in Figure 5a. A narrow energy range is used for PES of Au_6710_ and Au_7595_ to detail the finer features of the upper range of the energy landscape, at the expense of minima saturation. b) Rotational energy profiles for the same WK clusters at the sites A, B, C, A', B', and C' and midway points AB/2, BC/2, CD/2, A'B'/2, B'C'/2, and C'D'/2. Zero energy corresponds to the global minimum.

**Figure** **7**a reports the PES of WK Au clusters consisting of 157, 233, 6710, and 7595 atoms along the directions AD and A′D′ depicted in Figure 5a. The contact layers in Au_157_ and Au_6710_ have edges alternating two different lengths, and for this kind of contacts we locate the global minimum along A′D′. In contrast, Au_233_ and Au_7595_ are examples of hexagonal contact with equally sized edges, and for them we locate the global minimum along AD. For this reason, Figure 7a reports the PES along A′D′ for Au_157_ and Au_6710_ and along AD for Au_233_ and Au_7595_. Complementary PES along AD for Au_157_ and Au_6710_ and A′D′ for Au_233_ and Au_7595_ are shown in Figure S5 in the Supporting Information.

It is instructive to compare the PES with the fully relaxed energy data of for the WK Au_157_ cluster at locally stable configurations discussed earlier. The local minima reported in Table 2 are found at R30, ±7.2° and ±15.3° in the case of fully relaxed WK Au_157_. We retrieve these same local minima also from the PES, namely at ±30° (along A′D′), ±7° (along AD), and ±15° (along AD). The small deviations between the fully relaxed minima and those of the PES (e.g., 7.2° vs. 7°) are expected, since to obtain the PES we scan the orientations in steps of 1°, and atomic relaxation only involves the *z* coordinates. Based on this accord, we are confident that the local minima predicted by this procedure are very similar to those obtained by full relaxation. The entire PES is thus likely to provide a fair quantitative indication of the corrugation energy landscape that a deposited nanoparticle would encounter when it is made to translate/rotate across the graphene surface.

A closer look at the PES shows that for all the WK Au clusters,local minima are located at the sites A, B, C, and D or at the midway sites AB/2, BC/2, and CD/2. Hence, it is instructive to plot the orientational energy at these sites to study the local minima by correlating with the PES. Figure 7b reports the variation in energy as a function of the cluster orientation (pure rotation) at the sites A, AB/2, B, BC/2, C, and CD/2 and at the corresponding dashed points (dashed lines) for Au_157_ and Au_6710_. The global minima are −30° (at sites A′, B′, and C′ quasi‐degenerate within 0.2 meV), ±30° (R30, A), −30° (at A′, B′, and C′ quasi‐degenerate within 0.5 meV), and ±30° (R30 at A, B, and C quasi‐degenerate within 0.1 meV) for Au_157_, Au_233_, Au_6710_, and Au_7595_, respectively. Clearly, for our considered clusters, the orientation of the global minimum is always R30. For Au_157_, the other prominent local minimum is observed at ±7° (at B) which is roughly 1.5 meV higher in energy than the global minimum. There are three local minima roughly 1.5 meV higher than global minimum which are quasi‐degenerate within 0.2 meV in the case of Au_233_: ±30° (R30 @B, @C) and ±5° (@A). For the larger clusters, the energy landscape is mostly “flat” away from the minima. For this reason, large Au clusters would experience very weak barriers against diffusion once they move out of their global minimum at the optimal orientation. The PES also shows larger energy differences between the global minimum and the second lowest‐energy minimum at C, R0. The difference in energy relative to the global minimum is ≈50 meV for both clusters.

### 2.3 PES and Its Relation to Diffusion of Supported Au Clusters

From the previous section, it is evident that cluster angular alignment is a key parameter in the energy landscape. We now demonstrate this further using diffusion as a case study to understand the usefulness of the PES.

**Figure 8:**
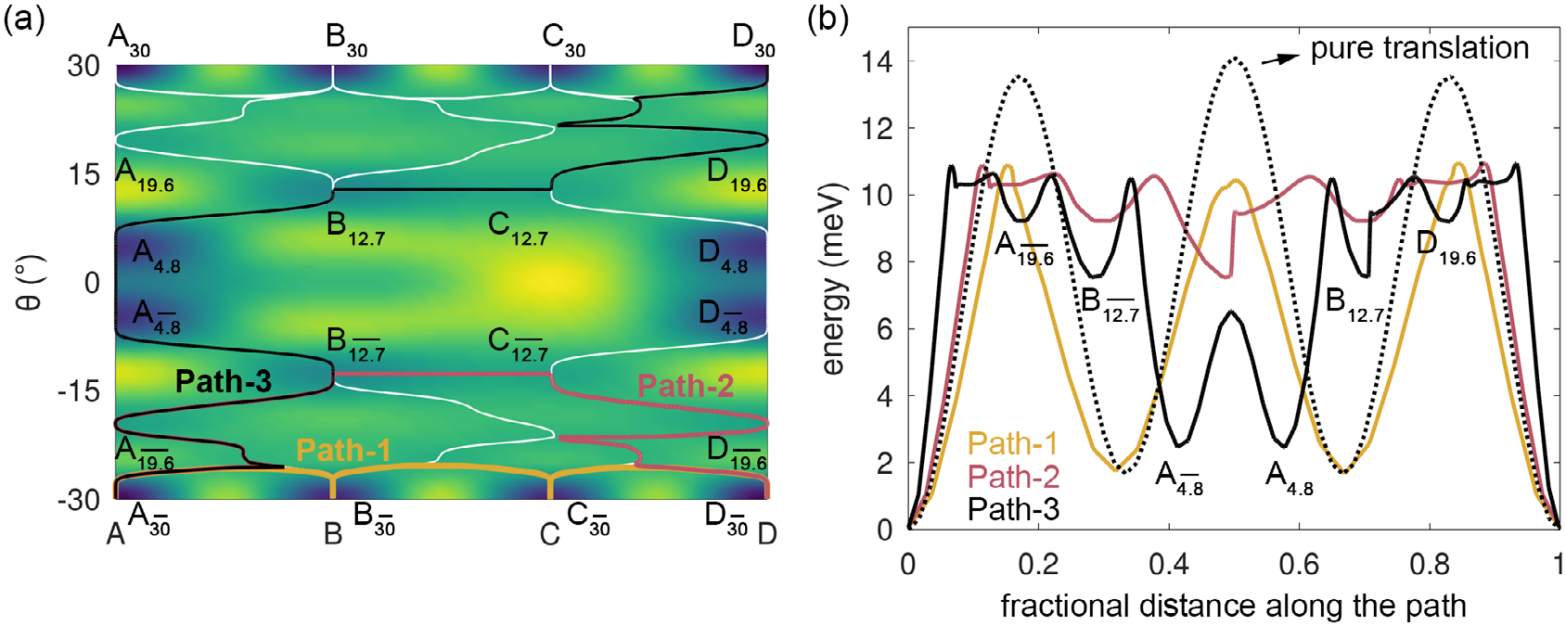
a) Energy surface (PES) of Au_233_ on three‐layer graphite as a function of rotation (*θ*) and translation (fractional distance) along the direction connecting two hollow sites A, D as shown in Figure 5a. White lines: minimum‐energy pathways between minima obtained by means of the string method.^[^ ^46^ ^]^ Three distinct A–D pathways (Path‐1—yellow, Path‐2—red, and Path‐3—black) are superimposed on the white lines. b) Energy along Path‐1, Path‐2, and Path‐3. The black dotted line is the energy of pure translation from A to D in R30 orientation.

**Figure** **8**a reports the PES of Au_233_ on 3L graphite. indicating the various local minima with the exact orientation angles extracted from full relaxations (see Figure S7 in the Supporting Information). The local minima are denoted as X_*θ*_, with X indicating the (A, B, or C) site and *θ* the cluster orientation. To move from one hollow site to an adjacent hollow site (A to D), the Au cluster can adopt one of several possible pathways. The simplest option is pure translation (fixed *θ*) from A_−30_ to D_−30_ (or equivalently, from A_+30_ to D_+30_). The energy profile along for this pure translation path is reported as a dashed line in Figure 8b: the resulting barrier is 13.54 meV. However, the Au cluster can encounter a lower barrier by changing its orientation away from R30. Using the string method,^[^ ^46^ ^]^ we identify three distinct pathways (Path‐1, Path‐2, and Path‐3, which involve rotation combined with translation. For all these three paths, reported in different colors in Figure 8a, the barriers are lowered to 10.9 meV, as indicated in the energy curves of Figure 8b. In practice, the Au clusters are expected to diffuse thermally through simultaneous rotation and translation, which may incidentally result in taking advantage of this barrier lowering.

To understand the diffusion mechanism in the context of the PES, we carry out a diffusion simulation of Au_233_ on a fully mobile 3L graphite at T=100 K (further details are provided in Section 4). The temperature is selected in order to have $k_{B}T$ similar to the discussed energy barrier against diffusion.

**Figure 9:**
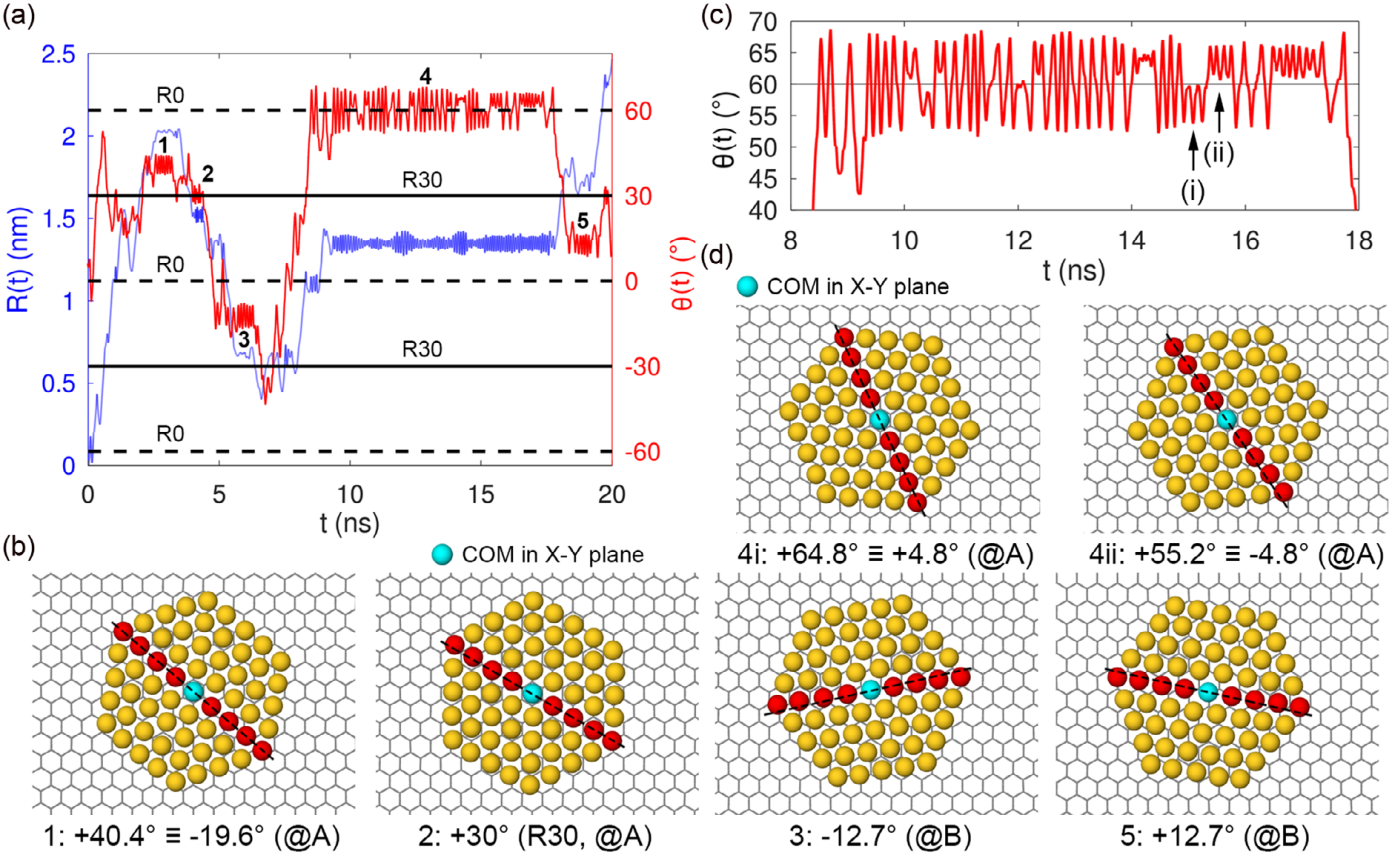
a) Displacement away from the initial position R(t) and orientation θ(t) of Au_233_ during an example of diffusion simulation at T=100 K. b) Representative snapshots of four pinning events (1, 2, 3, and 5 marked in (a)) along with the information of the local‐minimum orientation angle (dashed line) and the site location. c) Detail of the orientation of Au_233_ during the long pinning event 4 around 60°, showing the oscillations in the angular double well corresponding to the two minima at A with θ=±4.8°. Subregions (i) and (ii) are on opposite sides of the R0 line and represent vibrations in either well. d) Representative snapshots showing the cluster located in either angular well, with the equilibrium angles marked by dashed black lines. For clarity, in all snapshots only the interfacial Au layer is displayed.

**Figure** **9**a reports the distance R(t) from the starting point and the orientation θ(t), for a 20 ns simulation of the diffusion of Au_233_ on 3L graphite. As expected, the Au_233_ cluster undergoes simultaneous translation and rotation. Both R(t) and θ(t) exhibit pinning events guided by the PES. Five pinning events are marked 1–5 along the diffusive trajectory. These events occur at sites A or B, with orientations consistent with the minima of the PES. During the pinning events, both location and orientation are locked, with small‐amplitude oscillations near a minimum. These local oscillations are always present in both orientation and location. All the pinning events correlate well with the local minima observed in the PES. Events 1 and 2 occur at A sites, with θ=+40.4°≡−19.6° and θ=+30°, respectively. Events 3 and 5 occur at B sites, with θ=−12.7° and θ=+12.7°, respectively. Figure 9b reports representative snapshots during these pinning events. Event 4 is particularly interesting, since it is much longer than the other pinning events and involves oscillations between twin local minima at the A location and θ=±4.8°. The saddle point at R0 separates these twin minima with a shallow barrier of ≈4 meV. In this simulation, they are encountered at 60°±4.8°. Figure 9c reports the detail of the cluster orientation during event 4. We see two kinds of oscillations: oscillations within a single local minimum, with examples marked (i) and (ii), with a few degrees amplitude, and “tunneling” oscillations in the double well, crossing the R0 line due to their broader amplitude. The longer pinning time is likely due to the larger orientation range in the double well (evident from the higher amplitude) as opposed to the other minima, generating a substantial entropic advantage over single wells. Figure 9d displays representative snapshots of the configurations in the twin local minima on either side of 60°. Although the PES focuses on the line AD alone, and with rigid graphite, the pinning events of a realistic simulation correlate very well with the minima predicted along this direction. This is due to the fact that the lowest‐energy minima are located at the sites A, B, C, or D. Indeed, extensions of the PES to other positions on the surface (see Figure S8 in the Supporting Information) confirm that the lowest‐energy minima are located at either the hollow sites or the atop sites.

Based on the diffusion simulation, it is clear that the pinning events during the diffusion of Au cluster on graphite are guided by the PES. In effect, information of the pinning sites is already available in the PES even before carrying out diffusion simulations. In addition, the PES also explains why combined roto‐translational trajectories are favored against pure rotation or translation. In our relatively short diffusion simulation, not all local minima in the PES are visited. Exploring all minima in detail would require far longer simulations, which could accumulate a statistically significant number of pinning events. We defer a detailed study of the diffusion of Au clusters on graphite to future work.

We have evaluated the PES by locking the Au atomic positions in the xy plane while allowing relaxation in the *z* direction. Locking the orientation in this manner does not produce any artifacts as the local minima from full relaxation (without any orientation locking) are identical to the local minima predicted from the PES. Along with this, we scan the PES in specific directions (either AD or A′D′). Although a 3D PES covering all positions within a unit cell and rotations is desirable, we find that the PES we have calculated is sufficient to study the prominent local minima. We made the following checks to confirm this: 1) during diffusion, the cluster pinning sites match the local minima on the PES, and 2) scanning the PES in other directions (see Figure S8 in the Supporting Information) still results in the same set of lowest‐energy minima.

### 2.4 Comparison of Interaction Models

In this section, we compare different interaction models to understand how they affect the local minima and the energy landscape. The structure and dynamics of the gold clusters on graphite are determined by three interactions: Au—Au, Au—C, and C—C. For the C—C interactions, we stick to a combination of a reactive empirical bond order (REBO) potential^[^ ^47^ ^]^ and interlayer Kolmogorov–Crespi potential,^[^ ^48^ ^]^ which is considered highly reliable for graphite and is irrelevant anyway to any simulation carried out with a frozen substrate. We verify the robustness of our results adopting different models for the Au—Au and Au—C terms. For Au—Au, our default model interaction is the Gupta‐type potential,^[^ ^49^ ^]^ and we compare it with an embedded atom method (EAM) potential.^[^ ^50^ ^]^ For the Au—C term, our default model is the SAIP,^[^ ^32^ ^]^ which we compare to the de facto standard of the field so far^[^ ^30^, ^39^, ^51^, ^52^, ^53^ ^]^ a suitably tuned combination of LJ potentials.^[^ ^54^ ^]^ In this way, we explore the effect of inverting the energy ordering of the atop and hollow sites, as discussed in Section 1. We then compare three combinations of interaction models: 1) our default Gupta + SAIP, 2) EAM + SAIP, testing the intra‐cluster interactions, and 3) Gupta + LJ, testing the Au—C interaction. Details of the various interaction models are provided in Section 4.

**Figure 10:**
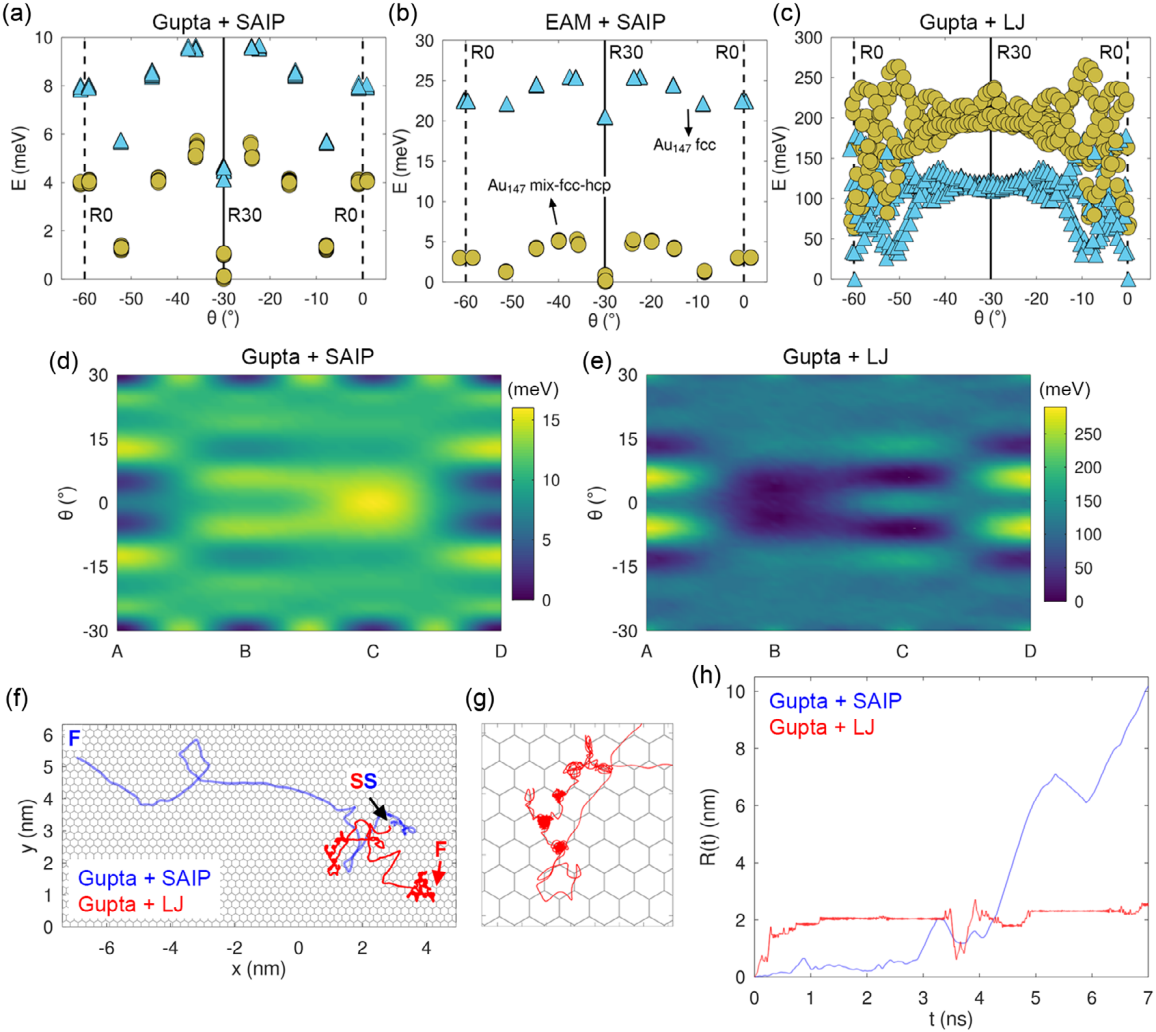
a–c) Energy of fully relaxed local‐minima structures of Au_147_ on 1L graphene belonging to fcc and mix‐fcc‐hcp motifs at the sites A, B, C, A′, B′, and C' according to three interaction models: a) the standard Gupta + SAIP used through this article, b) EAM + SAIP, and c) Gupta + LJ. d,e) Comparison of the PES of WK Au_233_ on 3L graphite obtained with d) Gupta + SAIP and e) Gupta + LJ interaction models. The A–D sites are shown in Figure 5a. f) Comparison of diffusion COM trajectories of WK Au_233_ on 3L graphite at T=300 K, simulated for 7 ns with the two models. *S* = starting position, pointed at by a black arrow; *F* = final positions. g) Zoomed initial section of the Gupta + LJ COM trajectory. h) Comparison of the displacement R(t) away from the starting position S for these same trajectories.

As reported in Figure S9 in the Supporting Information, the three interaction models predict fairly close energy differences between the best structures belonging to fcc, mix‐fcc‐hcp, Dh, and other motifs. In contrast, the orientation energy landscape exhibits significant differences. In **Figure** **10**a–c, the three interaction models are assessed against the orientation energy of the same Au_147_ clusters shown in Figure 5a,b at the sites A, B, C, A′, B′, and C′. Gupta and EAM are qualitatively similar, in that the energy ordering of the local minima is the same, with EAM potential placing the fcc structure even higher up in energy (note the different energy scale of panels a and b). In contrast, changing the Au—C interaction has a more relevant effect. To begin with, the lowest‐energy structure moves to R0 orientation with Gupta + LJ as opposed to R30 according to Gupta + SAIP and to EAM + SAIP.

In addition, there is significant difference also in the equilibrium vertical spacing between the Au contact layer and graphene. In the case of mix‐fcc‐hcp structure, the Au—C distance is ≃3.4 Å for EAM + SAIP and for Gupta + SAIP, in substantial agreement with recent DFT simulations^[^ ^55^ ^]^ (3.2 Å to 3.4 Å for Au_*N*_, N=5 to 19). In contrast, Gupta + LJ predicts an unphysically short distance ≈2.6 Å.

To better characterize the quantitative energy differences, we examine two quantities: $\varepsilon_{c}^{*}$ (defined as the difference between maximum‐energy and minimum‐energy orientations in Figure 10a–c) and the adhesion energy. Here, we note that $\varepsilon_{c}^{*}$ gives a sense of the relative corrugation across the models; however, it is not a true corrugation since it measures only the energy difference between local minima and not the full corrugation of the PES. We refer to $\varepsilon_{c}^{*}$ as relaxed corrugation. The adhesion energy ($\varepsilon_{a}$) is defined as follows:

(1) $$\varepsilon_{a}=\frac{E_{\text{Au}}+E_{C}-E_{\text{Au}+C}}{A_{\text{Au}-C}}\;$$

Here, $E_{\text{Au}+C}$ is the total energy of the fully relaxed Au cluster interacting with the C substrate. $E_{\text{Au}}$ and $E_{C}$ are the energies of the Au cluster and of the C substrate, obtained after detachment of the cluster from the substrate and local minimization of both cluster and surface separately. $A_{\text{Au}-C}$ is the interfacial area.

For calculating the interfacial area, first, the number of triangles formed by the nearest neighbor Au atoms are calculated. The interfacial area is then calculated by summing up the area of these triangles assuming them to be equilateral with the edge length equal to the mean Au—Au bond length in the Au contact layer. The values of $\varepsilon_{c}^{*}$ and $\varepsilon_{a}$ are reported in **Table** **3**.

**Table 3:** Corrugation energy ($\varepsilon_{c}^{*}$) and adhesion energy ($\varepsilon_{a}$) of the lowest‐energy structure on graphene for fcc and mix‐fcc‐hcp motifs.

Structure	Gupta + SAIP		EAM + SAIP		Gupta + LJ	
	$\varepsilon_{c}^{*}$	$\varepsilon_{a}$	$\varepsilon_{c}^{*}$	$\varepsilon_{a}$	$\varepsilon_{c}^{*}$	$\varepsilon_{a}$
	[meV]	[mJ m^−2^]	[meV]	[mJ m^−2^]	[meV]	[mJ m^−2^]
fcc	5.59	529	5.21	523	178.69	808
mix‐fcc‐hcp	5.72	536	5.32	543	202.02	821

We see that the LJ interaction model predicts a larger adhesion energy compared to SAIP. But, more importantly, we find a dramatic difference in $\varepsilon_{c}^{*}$ of LJ compared to SAIP: more than one order of magnitude, noticeable especially in the scale of Figure 10c compared to panels (a,b). This far higher and most likely unrealistic corrugation produced by the LJ model is mainly to be attributed to the underestimation of the Au—C distance, compared to a more realistic DFT estimation, also reproduced by the SAIP potential.

Figure 10d,e compare the PES of WK Au_233_ on 3L graphite obtained with the standard Gupta + SAIP and with the Gupta + LJ. Interestingly, the two PES appear approximately negative images, with the high‐energy regions of Gupta + SAIP turned to low‐energy regions for Gupta + LJ. The latter force field places the global minimum at site B, with θ=±4°, with other prominent minima located at C with θ=±6°, ≈4 meV higher than global minimum, and at A with θ=±14°, ≈26 meV above the global minimum. Besides this inversion, all Gupta + LJ barriers are one order of magnitude larger than those of Gupta + SAIP due to the larger corrugation discussed eearlier. As a result of the different corrugation, the same Au cluster at the same temperature will diffuse at a much faster rate when described by the Gupta + SAIP interaction. A diffusion simulation driven by the Gupta + LJ force field will exhibit longer pinning time in comparison with Gupta + SAIP.

In order to test the expectation mentioned earlier based on the PES, we carry out two comparative 7 ns diffusion simulations of Au_233_ at T=300 K, driven by both interaction models. Figure 10f shows the trajectories of the cluster COM in the xy plane. Figure 10h reports the displacement R(t) away from the starting position S. It can be immediately noticed that at room temperature, the Au_233_ cluster diffuses to longer distance with hardly any pinning by the Gupta + SAIP weak energy barrier. In contrast, with Gupta + LJ, we observe a significant proportion of pinning along with less frequent cluster sliding. The cluster remains pinned at the double well representing the global minima at B for θ=±4° which encounter a barrier of ≈26 meV to cross into one another.

As documented in Figure S10a in the Supporting Information, two types of sliding events are observed: sliding between the sites B and C with the orientation executing small‐amplitude oscillations around R0 and sliding coupled with wide‐angle changes in orientation. Both these diffusive motions are recognizable in Figure 10g. Short sliding of type B→C or C→B occur with no change in orientation. Within the same figure, the initial section of a long sliding event can also be observed: this sliding event spans a significantly long distance, while the cluster rotates away from R0 by ≈240° and eventually locks into R0 again at a far away pinning position, as visible between 3 and 4 ns in Figure S10a in the Supporting Information.

These simulations emphasize that the mechanisms and rates of diffusion are significantly different for Gupta + SAIP and Gupta + LJ. In practice, due to a quite flat corrugation and low energy barriers, the Gupta + SAIP predicts hardly any pinning at T=300 K, with diffusion dominated by long ballistic flights. As a result, we anticipate that room‐temperature simulations of Au nanoparticles would take far longer to reach a linear diffusion regime (i.e., a mean square displacement increasing linearly with time) when simulated with Gupta + SAIP than if one would adopt the less realistic Gupta + LJ model, which exhibits linear diffusion based on just few ns of simulation, as reported in Figure S10b in the Supporting Information.

Our results clearly establish that the accuracy of Au—C interaction (SAIP vs. LJ) is crucial for studying Au clusters on graphitic substrates in comparison with Au—Au interaction (Gupta vs. EAM). Because of the contrasting adsorption site preferences, the significant difference between corrugation energy predicted by SAIP and LJ has broader implications for cluster sliding which is greatly influenced by corrugation felt by the cluster. In such scenarios, we expect SAIP, which has much better agreement with DFT,^[^ ^32^ ^]^ to give qualitatively accurate trends compared to LJ which is currently the most common interaction model for Au—C.

## 3 Conclusions

In summary, we have carried out a systematic study to understand the structural arrangement and energy landscape of Au nanoclusters grown on graphene and graphite using a state‐of‐the‐art model for the Au—C interaction, SAIP.^[^ ^32^ ^]^ In contrast to gas‐phase clusters, on graphene/graphite Au clusters adopt geometries which are based on stacked close‐packed layers, i.e., (111) epitaxy, either as fcc or defective mix‐fcc‐hcp structures. Noncrystalline motifs (icosahedron and decahedron) that are common in unsupported clusters are completely absent because they lead to high strain at the Au—C interface. Based on this information obtained for a few relatively small clusters sizes, we generate larger energetically stable clusters using the WK construction.

On graphite and graphene alike, the energetics of a Au cluster depends on its position and orientation. Two main orientations relative to the C lattice are especially relevant: R0 and R30. The R0 orientation, with close‐packed ⟨110⟩ directions of the Au cluster aligned in the zigzag directions of graphene, leads to significant mismatch between the Au lattice and graphene which rapidly forms a moiré pattern as the size increases, often leading to local minima rotated a few degrees away from R0. In R30, the much smaller mismatch allows for pseudo‐commensurate arrangements for clusters up to ≈21 nm in lateral size. As a result, R30 turns out the best orientation for all Au clusters studied in this work, with several competing local minima occurring at various positions of the cluster COM on the C lattice.

Detailed information regarding not just the local minima but also the barriers in between is encoded in the PES, namely the potential energy as a function of the cluster COM position in the xy plane and angular orientation. Of this function of three variables, we provide 2D cuts along specific lines for Au clusters up to a size of 7595 atoms. The PES allows us to assess various minimum‐energy pathways for cluster sliding and diffusion. Analysis of Au_233_ on graphite reveals that pure translation or pure rotation have higher barriers for cluster sliding compared to pathways that involve simultaneous rotation and translation. The role of cluster rotation or “twisting” in further lowering the already weak barriers against translation agrees with the recent report of sudden and reversible friction changes during nanomanipulation experiments of Au nanoparticles on graphite.^[^ ^21^ ^]^ Using a sinusoidal corrugation energy model, it was rationalized that rotation of the particle is responsible for the observed friction fluctuations. Friction is directly related to the cluster sliding barrier and in our model (Gupta + SAIP) we find the same phenomena—cluster rotation combined with translation leads to pathways with reduced sliding barriers.

The PES will be useful in future investigations of cluster diffusion, and potentially for friction experiments too, in the line of refs. [39, 56, 57, 58, 59], but to be carried out in cryogenic conditions, due to the lower lateral barrier of Au—C, compared to previously investigated interfaces. The information of the pinning sites for diffusion is encoded in the PES in the form of local minima. Our diffusion simulations of Au_233_ on 3L graphite reveals several pinning events, exhibiting oscillations around the positions and orientations of the cluster which match precisely the local minima of the PES.

To test how these conclusions depend on the detailed microscopic interactions involved, we assess various models for the Au—Au and Au—C interactions, while keeping the C—C same interaction. For the Au—Au interaction, Gupta and EAM models are qualitatively similar with respect to orientation and energy ordering of the local minima. Both models predict R30 to be the best orientation. However, changing the Au—C interaction model has a significant effect. Compared to the accurate SAIP that we use for all simulations, we verified that a LJ Au—C interaction, sets the minima at R0, with a close Au—C approach and high lateral corrugation barriers >170 meV. Experiments are consistent with the fast room‐temperature diffusion of Au clusters on graphite, with pinning at surface defects only, never at the middle of a perfect terrace, in clear agreement with the SAIP model against the oversimplified LJ model.

## 4 Model and Methods

### Interaction Model

The Au—Au interactions are modeled by the Gupta potential,^[^ ^60^ ^]^ which is developed within the second‐moment approximation within the second‐moment approximation to the tight‐binding model (TBSMA).^[^ ^61^ ^]^ The potential parameters used in this work are given in ref. [49]. The Au—C interactions are modeled by SAIP. The form and parameters of this SAIP are given in ref. [32]; in particular, the cutoff distance of this interaction equals 16 Å. The C—C interactions are modeled by a combination of a second‐generation REBO potential^[^ ^47^ ^]^ and interlayer Kolmogorov–Crespi registry‐dependent potential.^[^ ^48^ ^]^

For comparison, we keep the same C—C interaction, and we consider an alternative EAM^[^ ^50^ ^]^ potential for Au—Au^[^ ^62^ ^]^ interaction and an alternative LJ potential for the Au—C interaction. The LJ Au—C parameters taken from ref. [54] are σ=2.74 Å and ε=22 meV, with a 7 Å cutoff length. We compare three combinations of Au—Au + Au—C interaction models: 1) Gupta + SAIP, 2) EAM + SAIP, and 3) Gupta + LJ.

### Parallel Tempering Molecular Dynamics (PTMD)

To determine the global and local equilibrium structures of Au clusters on graphene, we carry out MD combined with PTMD.^[^ ^36^ ^]^ In this method, we begin with several copies (or replicas) of the system, each of them thermostated at a different temperature. To promote an efficient sampling of the configuration space at lower temperatures, adjacent replicas are allowed to exchange configurations periodically.^[^ ^36^ ^]^ There are *M* replicas each at temperatures $T_{m}$ (m=1,2,3,…,M). The number of replicas is chosen to generate a ≈40% acceptance rate of replica swaps. All replicas are simulated within the canonical ensemble (NVT). We use a time step of 5 fs, and each PTMD simulation is carried out for 60 ns for Au_55_ and 75 ns for Au_147_. An exchange of a pair of adjacent replicas (*m* and *n*) is attempted every 125 ps which is either accepted or rejected according to probability given by the following Metropolis‐like criterion:

(2) $$p=\min\left(1,e^{-(E_{n}-E_{m})(\beta_{n}-\beta_{m})}\right)$$

where $\beta_{m}=1/(k_{B}T_{m})$ and $\beta_{n}=1/(k_{B}T_{n})$. The potential energies of the replicas *m* and *n* are $E_{m}$ and $E_{n}$ respectively. We carried out all the PTMD simulations in large‐scale atomic/molecular massively parallel simulator (LAMMPS).^[^ ^63^ ^]^ The number of replicas and detailed list of temperatures are provided in Section S11 in the Supporting Information.

After each swap attempt, at 50 and 100 ps of simulation, we store a snapshot from each of the replicas. Starting from each of these snapshots, we carry out a conjugate‐gradient minimization, leading to the nearest local minimum of the potential energy.

Based on the common neighbor analysis (CNA) signatures^[^ ^64^ ^]^ of all the atoms, each local minimum is classified into one of the six structure classes: 1) fcc, 2) mix‐fcc‐hcp (which consists of fcc with faults such as twins, stacking defects, or even entirely hexagonal close‐packed), 3) decahedron (Dh), 4) icosahedron (Ih), 5) *other* (crystalline and amorphous regions within the same structure), and 6) amorphous. This is done by first isolating the Au cluster from the graphene substrate after relaxation and then calculating the CNA signatures. In this classification, we adopt the same scheme adopted previously for unsupported Au clusters.^[^ ^40^ ^]^

In this way, we obtain the fraction of each structure class as a function of temperature that we report in diagrams such as Figure 2d. In order to estimate the error bar on the fraction of a structure class at each temperature (1200 configurations per temperature), we create shorter data sets of 200 configurations drawn randomly from the 1200 resulting in 6 sets. This process is repeated 10 times to create 60 data sets consisting of 200 configurations. The error in fraction of fcc and mix‐fcc‐hcp is calculated as the standard deviation from the 60 data sets.

### Global Optimization

We also employ basin hopping Monte Carlo (BHMC)^[^ ^35^, ^65^ ^]^ to search for the lowest‐energy structure of the Au clusters on rigid graphene. Each BH step involves a short high‐temperature MD run to change the shape of the cluster followed by local relaxation (energy minimization). All the BH searches are initialized with a random disordered Au structure. We consider Au clusters consisting of 49, 58, 119, and 157 atoms on graphene. At each size, we run five independent BH searches with at least 50 000 steps for Au_49_ and Au_58_ and 30 000 steps for Au_119_ and Au_157_.

### WK Construction

PTMD simulations have shown that Au clusters adopt fcc‐based structures, i.e., (111) layers stacked either following the fcc or the hcp rule, or with stacking faults. We did not find any other motifs (Ih and Dh) which in vacuum are energetically close to the fcc structures. Hence, for the WK construction,^[^ ^37^, ^38^ ^]^ we consider only the fcc motifs with (111) epitaxy. Two key parameters dictate the overall shape of a Au cluster supported on C:

(3) $$\sigma =\frac{\gamma_{111}}{\gamma_{100}},\;\tau =\frac{\varepsilon_{111}}{\gamma_{111}}\;$$

Here, $\gamma_{100}$ and $\gamma_{111}$ are the surface energies of bulk Au. For Au described by the Gupta potential used in the current work, these quantities are $\gamma_{100}=527.7$ mJ m^−2^ and $\gamma_{111}=444.2$ mJ m^−2^, yielding a *σ* value of 0.84. For evaluating $\varepsilon_{111}$, we initially considered regular truncated octahedron (RTO) on graphene up to a maximum size of 17561 atoms. The adhesion energy for Au_17561_ on graphene is 442 mJ m^−2^. Our analysis shows that $\varepsilon_{111}$ increases sharply with the reduction of the interface area (see Figure S11 in the Supporting Information). For the WK construction, we adopt $\varepsilon_{111}=440$ mJ m^−2^ (≈ adhesion energy of the largest RTO Au_17561_), corresponding to τ=0.99. A comparison of the global minima and the WK shapes indicates that this choice yields reasonably correct cluster shapes (see Section S12 in the Supporting Information for further details). Once these parameters are fixed, we follow the recipe provided in ref. [38] for the (111) epitaxy and generate WK structures of different sizes.

### Diffusion Simulations

Keeping the computational cost in mind, we select a relatively small Au cluster consisting of 233 atoms diffusing on graphite. The Au—C interaction used in the current work has a cutoff of 16 Å. Given the distance between the Au contact layer to the top most C layer (>3 Å) and inter layer C—C distance (3.3716 Å), at least six carbon layers are required to simulate a semi‐infinite graphite substrate. However, the PES of 1L graphene, 3L graphite, and 6L graphite are nearly identical with regard to the local minima and the quantitative energy values (see Section S6 in the Supporting Information for further details). Here, we note that SAIP potential overestimates the adhesion energy of 3L Au—graphene interface,^[^ ^32^ ^]^ and hence PES of 1L and 3L look very similar. Based on this, it is sufficient to use 3L graphite for the PES and for diffusion simulations.

The bottom C layer is fixed, and the other two C layers are mobile. First, temperature is equilibrated to the desired value by means of a NVT Langevin thermostat applied for 1 ns to the intermediate and top C layers and to the Au cluster. After this, the thermostat is switched off and the time evolution proceeds according to NVE ensemble. All simulations are carried out in LAMMPS^[^ ^62^ ^]^ using a 1 fs time step. The COM coordinates are saved every 0.25 ps, and configurations are saved every 10 ps during diffusion. OVITO^[^ ^66^ ^]^ is used to analyze the configurations saved during diffusion.

## Conflict of Interest

The authors declare no conflict of interest.

## Supporting information

Supplementary Material

## Data Availability

The data that support the findings of this study are available from the corresponding author upon reasonable request.
