# Supplementary material for: Gold Clusters on Graphene/Graphite—Structure and Energy Landscape
Source: Small Sci. 2024 Aug 12;4(9):2400078. doi: 10.1002/smsc.202400078 (PMC11935057; doi:10.1002/smsc.202400078)
Supplement: Supplementary file 1 — Supplementary Material [file SMSC-4-2400078-s001.pdf]

Supplementary Information
for
Gold clusters on graphene/graphite – structure and energy landscape

Manoj Settem† *Melisa M. Gianetti*† *Roberto Guerra* *Nicola Manini** *Riccardo Ferrando** *Alberto Giacomello*

Manoj Settem, Alberto Giacomello
Dipartimento di Ingegneria Meccanica e Aerospaziale, Sapienza Università di Roma, via Eudossiana 18, 00184 Roma, Italy
Melisa M. Gianetti
Institutt for maskinteknikk og produksjon, NTNU, Richard Birkelands vei 2B, 7034 Trondheim, Norway
Roberto Guerra
Center for Complexity and Biosystems, Department of Physics, University of Milan, via Celoria 16, Milano 20133, Italy
Nicola Manini
Dipartimento di Fisica, Università degli Studi di Milano, Via Celoria 16, Milano 20133, Italy
Email Address: nicola.manini@fisica.unimi.it
Riccardo Ferrando
Dipartimento di Fisica dell'Università di Genova and CNR-IMEM, via Dodecaneso 33, 16146 Genova, Italy
Email Address: ferrando@fisica.unige.it
† These authors contributed equally to this work.

S1 Structure of mix-fcc-hcp vs. decahedron

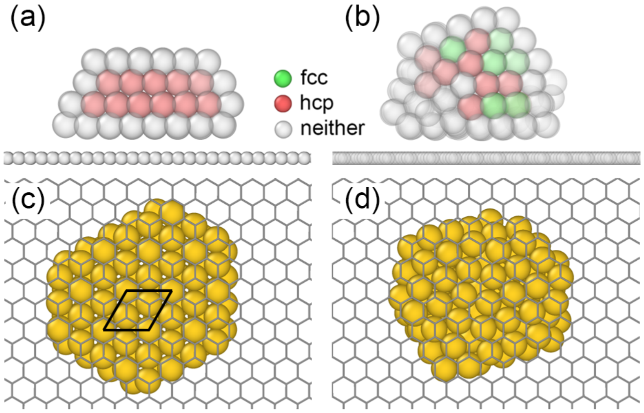

Figure S1: Side and bottom views of two locally stable structures of the Au_{147} cluster on graphene: (a,c) the global minimum, with mix-fcc-hcp, R30 orientation; (b,d) a decahedron (Dh). This Dh structure is ~ 3 eV higher in energy than the global minimum.

Figure S1 shows a comparison of the Au_{147} clusters having mix-fcc-hcp (global minimum) and decahedron (Dh) structures. In the mix-fcc-hcp structure, the Au layer in contact with the C lattice in R30 orientation is pseudo-commensurate, and relatively flat. The Dh structure has its decahedral axis approximately parallel to the Au-C interface. Hence, to maintain a reasonably flat interface, the two sub-units at the lower left are distorted. As a result, the arrangement of the Au atoms in the contact layer is not optimal, resulting in a worse stability of Dh.

S2 Structure of Au_{55} clusters on graphene

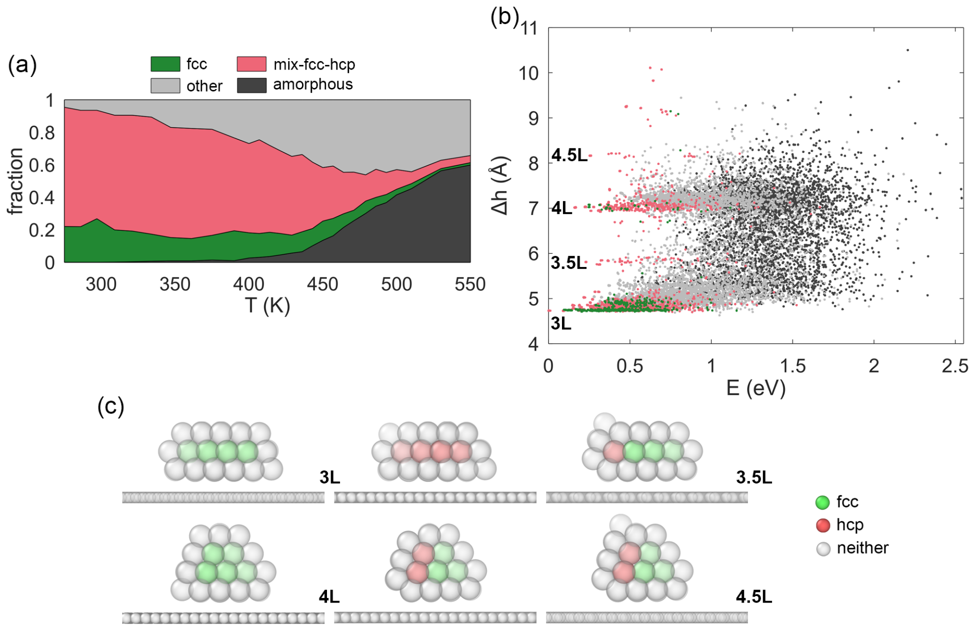

Figure S2: (a) Distribution of graphene-adsorbed Au_{55} structure types. (b) The Au_{55} cluster thickness Δh, correlated to the local-minimum potential energy. A point's color indicates the structure kind, with the same notation as in panel (a). (c) Side view of a few representative structures consisting of three (3L) or four (4L) layers. M.5L stands for structures exhibiting intermediate thicknesses. The left column reports fcc structures. The center and right columns report mix-fcc-hcp structures. Ball colors mark the local atomic coordination, as indicated in the legend. The scatter plot of Δh vs energy is constructed using 26,880 configurations sampled during PTMD. The increased proportion of *other* structures is immediately evident from the significant overlap of fcc/mix-fcc-hcp and *other* structures.

Figure S2 reports informations analogous to those of Fig. 2 of the paper, but for the Au_{55} cluster, rather than for Au_{147}.

S3 Configuration of Wulff-Kaischew (WK) Au_{157} in R30 orientation at the sites A, A′

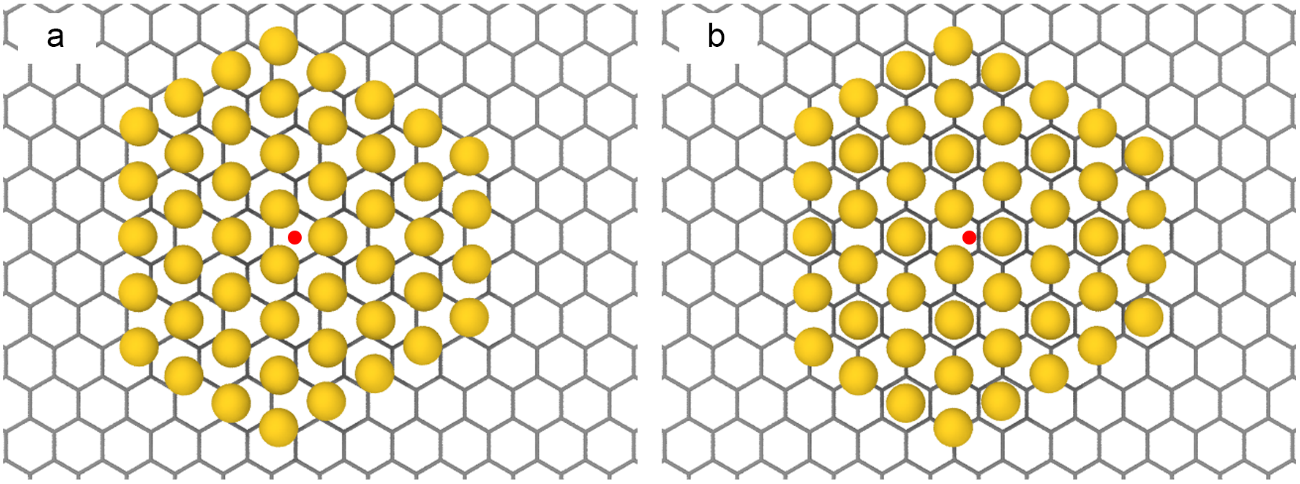

Figure S3: The cluster layer in contact with graphene for the WK structure of Au_{157} in R30 orientation at the site (a) A and (b) A′. The red circle marks the center of mass of the Au cluster.

Figure S3 compares the structure of the Au contact layer of the WK structure of the Au_{157} cluster at the competing sites A and A′. When the center of mass (COM) is at the site A in R30 orientation, all the Au atoms sit near bridge sites (midpoint of C-C bond) (Figure S3a). A shift by (1/3) of the unit cell vector along the horizontal direction leads to the configuration shown in Figure S3b, where the Au atoms sit either near the atop or the hollow sites. According to our Au-C model interaction, the most favorable adsorption site is atop followed by bridge (0.27 meV higher than atop) and hollow (1.23 meV higher than atop). The configuration at A′ has 32 Au atoms at atop sites and 16 Au atoms at hollow sites (total 48 atoms in the Au contact layer). On the other hand, the configuration at A has 48 Au atoms near bridge sites. The configuration at A′ has a high number (32) of favorable atop sites along with a lower number (16) of unfavorable hollow sites while the configuration at A has a very high number (48) of intermediate-energy bridge sites. Analogous situations occur when comparing B with B' and C with C' sites. Our simulations show that in the R30 orientation the sites A′, B′, and C′ are energetically favorable compared to the sites A, B, or C, indicating that for such small pseudo-commensurate structures the site competition favors combinations of low-energy atop sites + high-energy hollow sites against all bridge sites.

S4 Calculation of the orientation angle

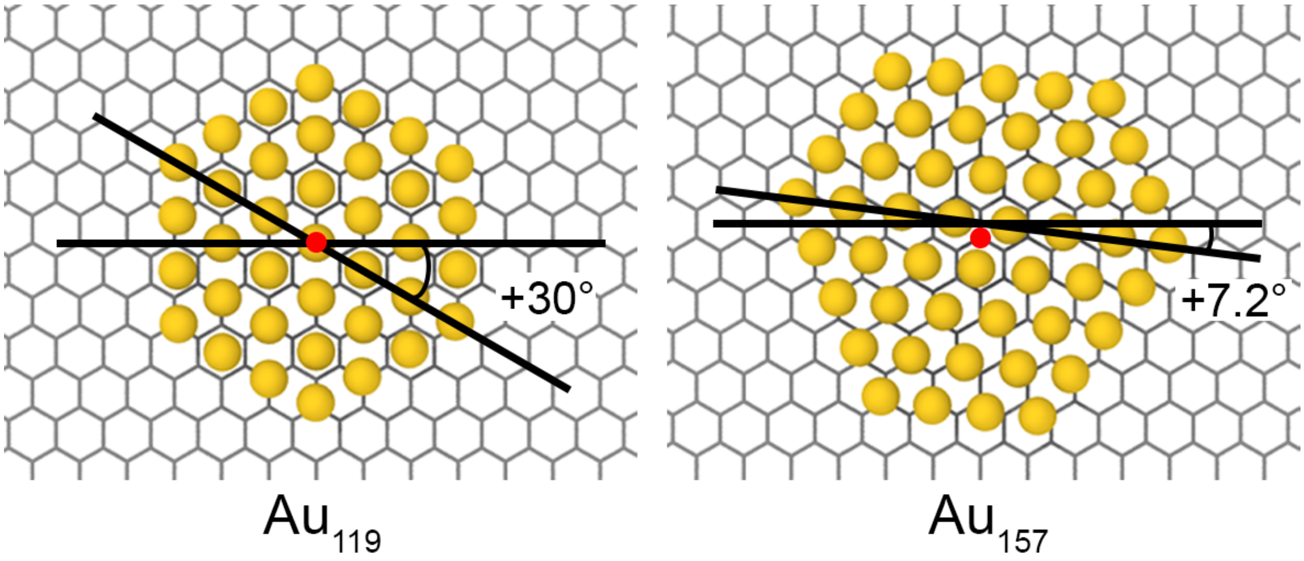

Figure S4: Au contact layer in WK Au_{119} and Au_{157} clusters. The red circle represents the center of mass of the Au cluster. The black line indicates linear fit of the row of atoms underneath the line.

The orientation θ of a Au cluster relative to the underlaying graphene is calculated as the angle formed by a row of nearest-neighboring atoms passing as close as possible to the center of mass (in the xy plane), with the substrate horizontal zig-zag direction. Figure S4 shows the Au contact layer for WK Au_{119} and Au_{157} and the substrate top layer. A row of atoms either passing through the center of mass (indicated by red circle) or close to it is chosen. The orientation of a linearly fit line (indicated by a black line in figure) through this row of atoms marks the orientation of the Au cluster. The slope of the fitted line relative to the x axis (the substrate zig-zag direction) is minus the tangent of the orientation angle θ, so that clockwise rotation is positive. In the above illustration, Au_{119} and Au_{157} have orientation $\theta = +30°$ (R30) and $\theta = +7.2°$, respectively.

S5 PES of Au_{157} and Au_{6710} along the direction AD

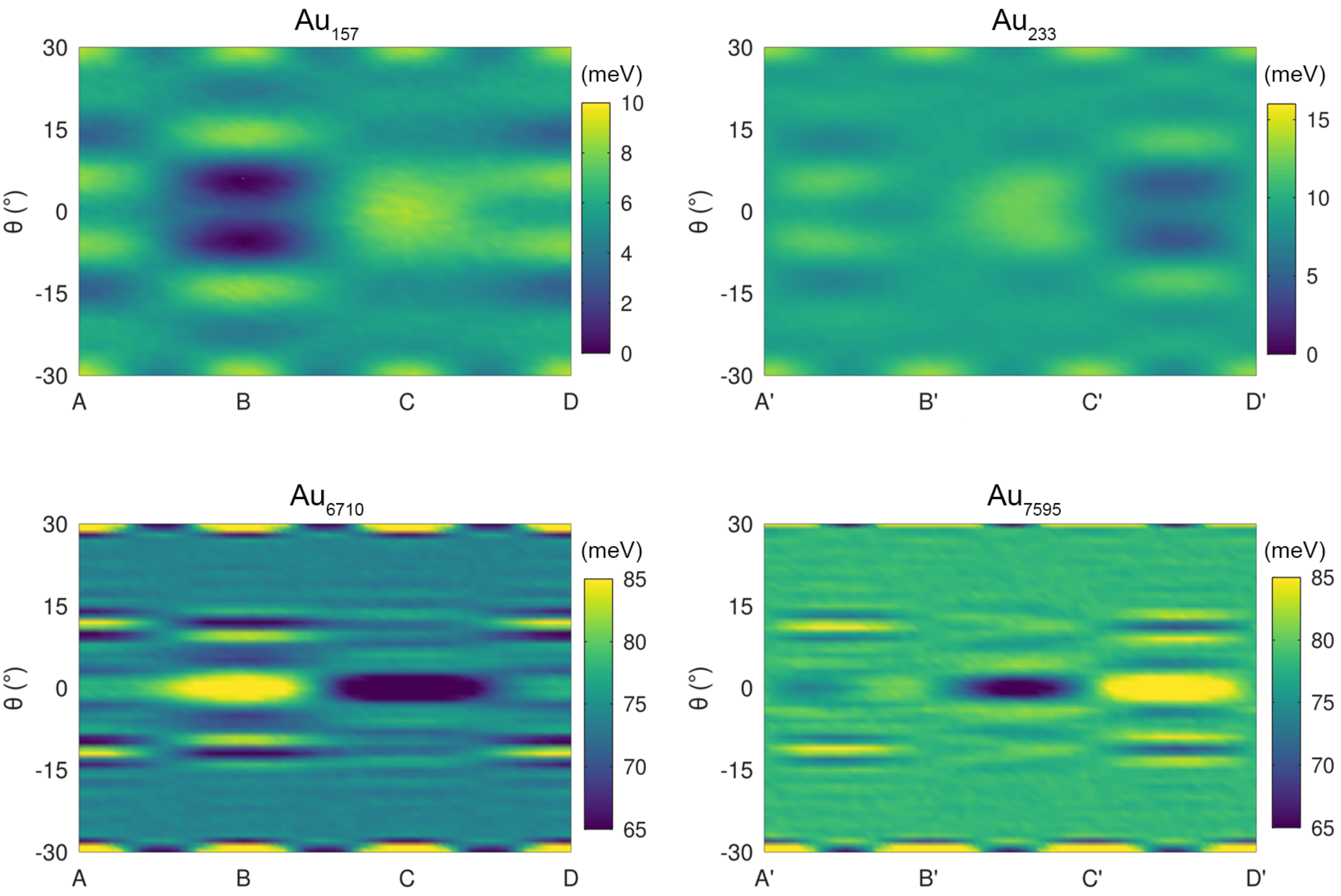

Figure S5: PES of WK clusters on graphene along the lines AD for Au_{157} and Au_{6710}, and along A′D′ for Au_{233}, Au_{7595}. A narrow energy range is used for PES of Au_{6710} and Au_{7595} to highlight the finer details in the energy landscape.

S6 PES of Au_{233} on 1, 3, and 6 layers of carbon

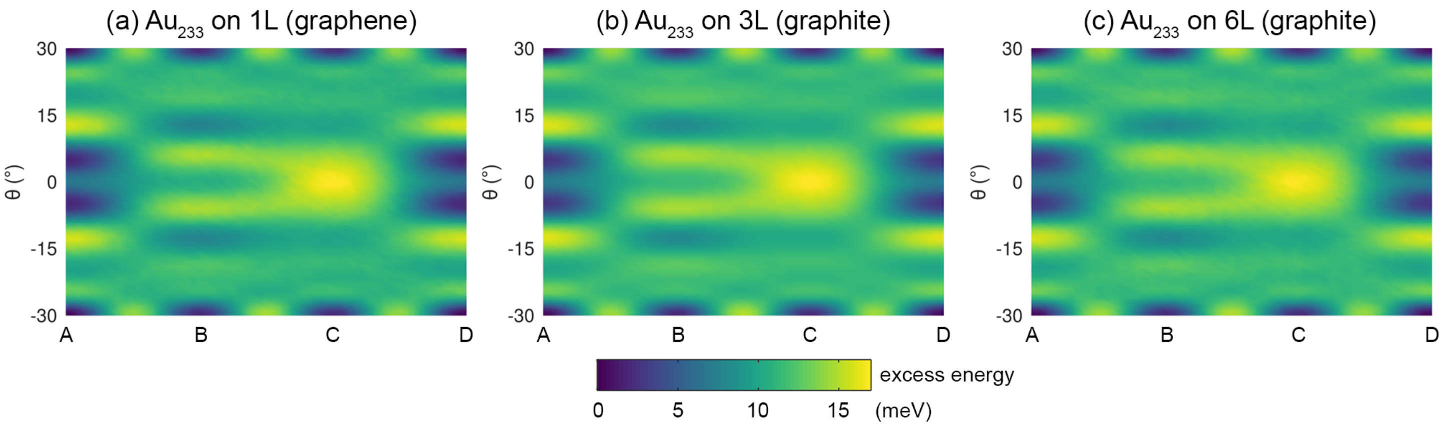

Figure S6: Comparison of the PES of WK Au_{233} on (a) 1L graphene, (b) 3L model for graphite, and (c) 6L model for graphite, all in the same energy scale of the excess energy relative to the minimum for each substrate.

The SAIP Au-C interaction has a cutoff distance of 16 Å. The distance between the contact layer pf Au clusters and the topmost C layer slightly exceeds 3 Å. The interlayer C-C distance in graphite modeled with the REBO + Kolgomorov Crespi (KC) interaction is 3.3716 Å. As a result, the top 5 layers of graphite lie inside the SAIP Au-C cutoff radius. Thus, to simulate accurately the cluster interaction with a semi-infinite graphite bulk, a priory at least six carbon layers need to be included.
To test if this thickness could be reduced, in Figure S6 we display the PES for the WK Au_{233} cluster deposited on 1-layer (1L) graphene, 3-layer (3L) graphite, or 6-layer (6L) graphite. Visually, the PESs for these three different substrates look nearly identical. In particular, there is hardly any difference in the positions of the local minima and barriers. Quantitatively, the overall corrugation amplitude for 1L, 3L, and 6L are 14.2 meV, 15.8 meV, and 16.1 meV are quite close, suggesting that even 1L produces fair semi-quantitative agreement with bulk graphite, and 3L is nearly quantitatively identical. Based on this analysis, in order to save computation cost, we adopt the 3L model of graphite to study the diffusion trends of Au clusters on graphite.

S7 Fully relaxed Au_{233} on 3L graphite

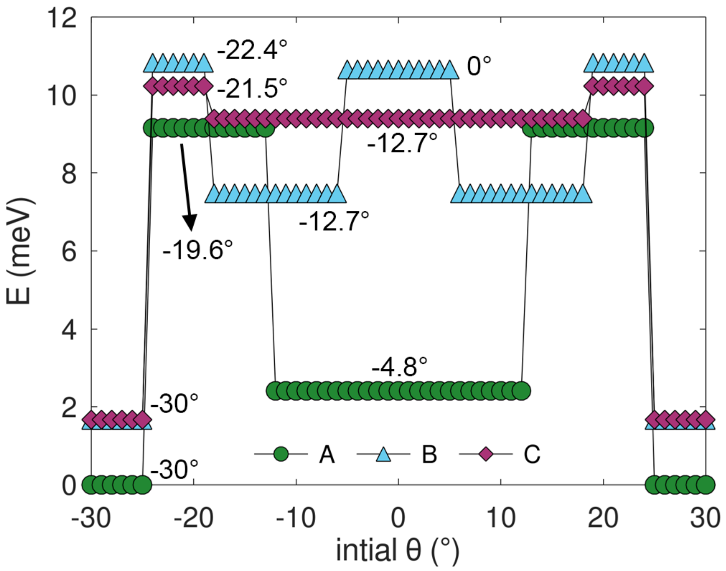

Figure S7: Energy (relative to the global minimum) of fully relaxed Au_{233} on 3L graphite as a function of the cluster's initial orientation, with the center of mass initially at the 3 high-symmetry sites A, B, C on the graphite surface. The final relaxed orientation angle is indicated near each "terrace".

To evaluate the precise orientation angles of the local minima of WK Au_{233} on 3L graphite, we carry out a full relaxation of the Au atoms on a rigid graphite substrate. With the cluster center of mass placed at the sites A, B, and C, we scan the angular range $-30°$ to $30°$ in steps of $1°$, because positive θ yields symmetrically equivalent results. Figure S7 reports the excess energy as a function of the initial orientation is shown. The final orientation at the end of the relaxation is indicated. Each "terrace" represents a single locally-stable orientational minimum, and the width of the terrace itself represents the angular basin of attraction of that minimum. The indicated final angles are evaluated according to the procedure described in section S4.

S8 PES of Au_{233} on 3L graphite along other directions

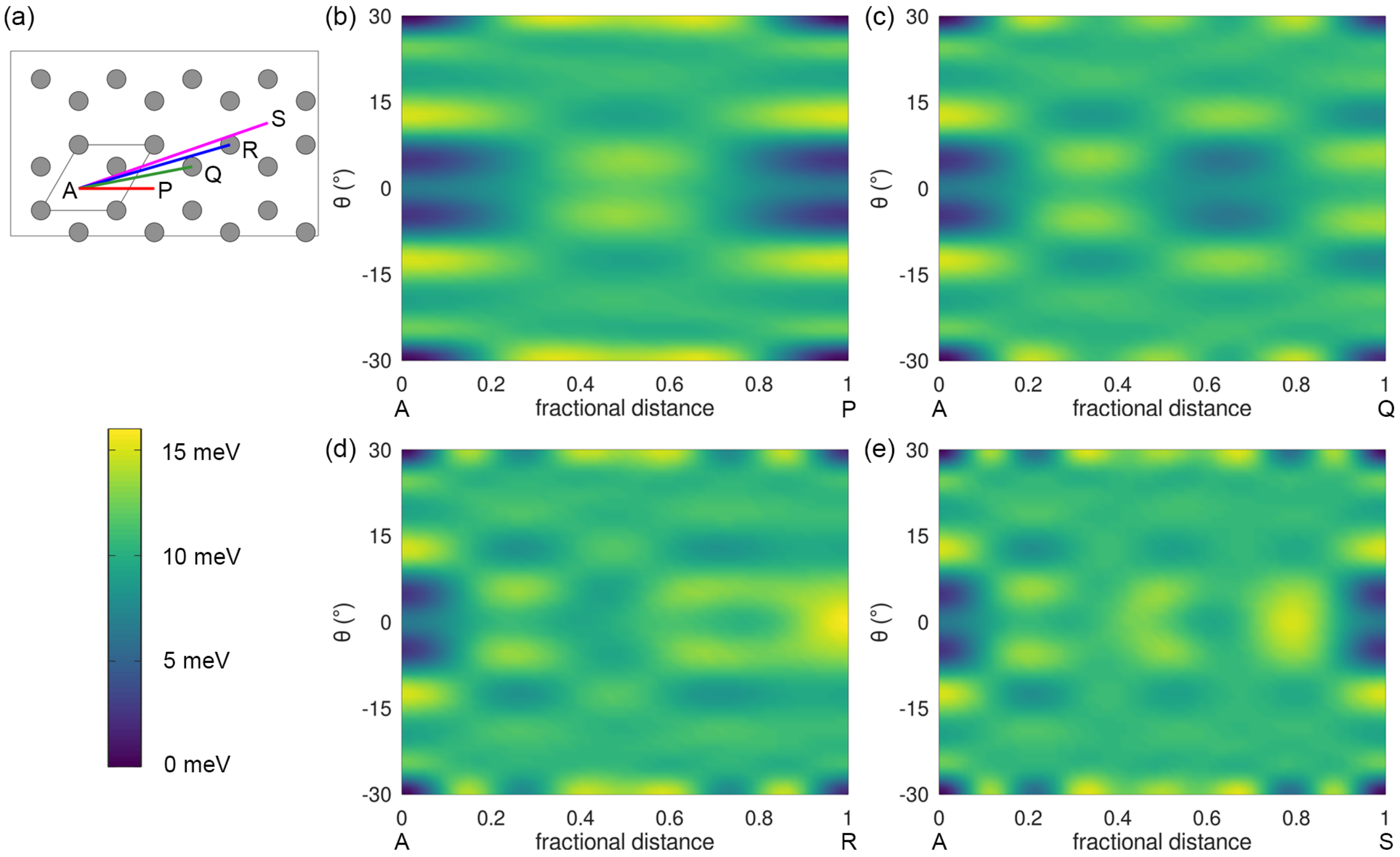

Figure S8: (a) Graphene lattice showing the various paths along which the rt-PES is computed for WK Au_{233} on 3L graphite. The PES evaluated as a function of the orientation θ and center-mass displacement along (b) AP, (c) AQ, (d) AR, and (e) AS.

S9 Comparison of different force fields

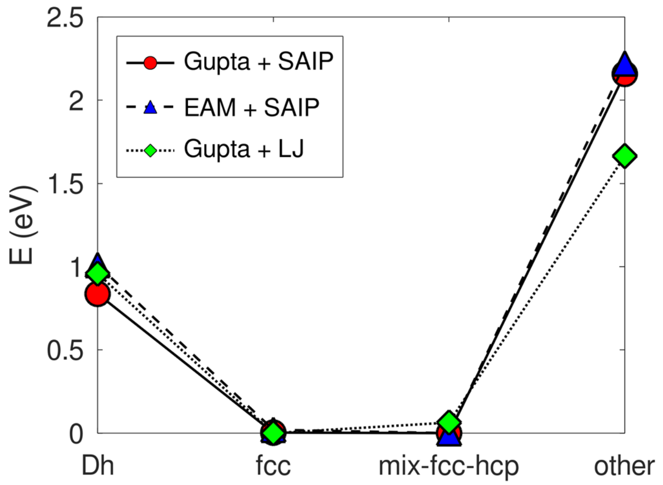

Figure S9: Comparison of the energy differences between the best relaxed structures of Au_{147} on graphene, classified according to the motifs discussed in the text, according to three interaction models: our default Gupta + SAIP (circles), EAM + SAIP (triangles), and Gupta + LJ (diamonds).

Figure S9 reports a comparison of the energetics for the best relaxed structures of the main kinds, as obtained with different model interactions.

S10 Diffusion with the Gupta + LJ model

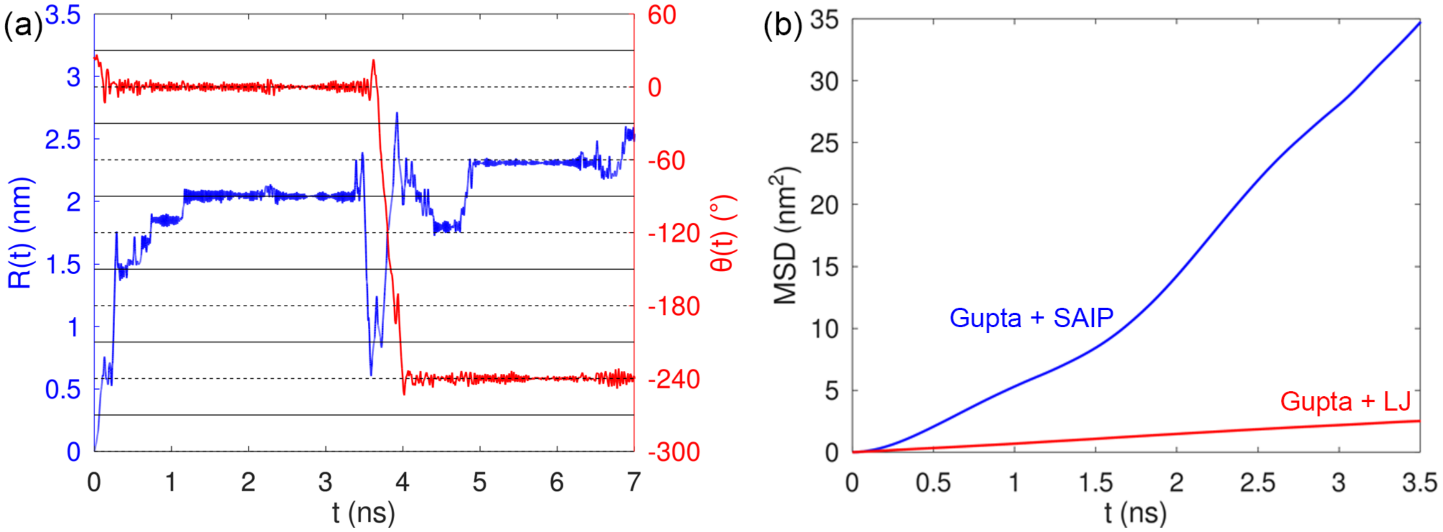

Figure S10: (a) Displacement $R(t)$ from the initial point and orientation $\theta(t)$ of a Au_{233} cluster in a simulated diffusion at $T = 300$ K, driven by the Gupta + LJ interaction model. Horizontal dashed lines and horizontal solid lines indicate R0 and R30 orientation respectively. (b) Comparison of the mean squared displacement (MSD) evaluated based on 7 ns simulated diffusion executed with the Gupta + SAIP and with the Gupta + LJ models, both at $T = 300$ K.

S11 Parameters of PTMD simulations

Table S1. Number of replicas and replica temperatures used for PTMD structural investigations of Au clusters on graphene, simulated based on the Gupta + SAIP model.

Metal cluster	# replicas	Replica temperatures (K)
Au_{55}	28	275, 286, 297, 309, 321, 334, 347, 361, 375, 390, 400, 407, 414, 421, 429, 436, 443, 450, 457, 464, 471, 479, 486, 493, 500, 510, 530, 550
Au_{147}	24	300, 316, 333, 351, 370, 390, 410, 432, 456, 480, 500, 516, 532, 548, 564, 580, 596, 612, 628, 644, 660, 680, 714, 750

S12 Adhesion energy of Au clusters on graphene/graphite

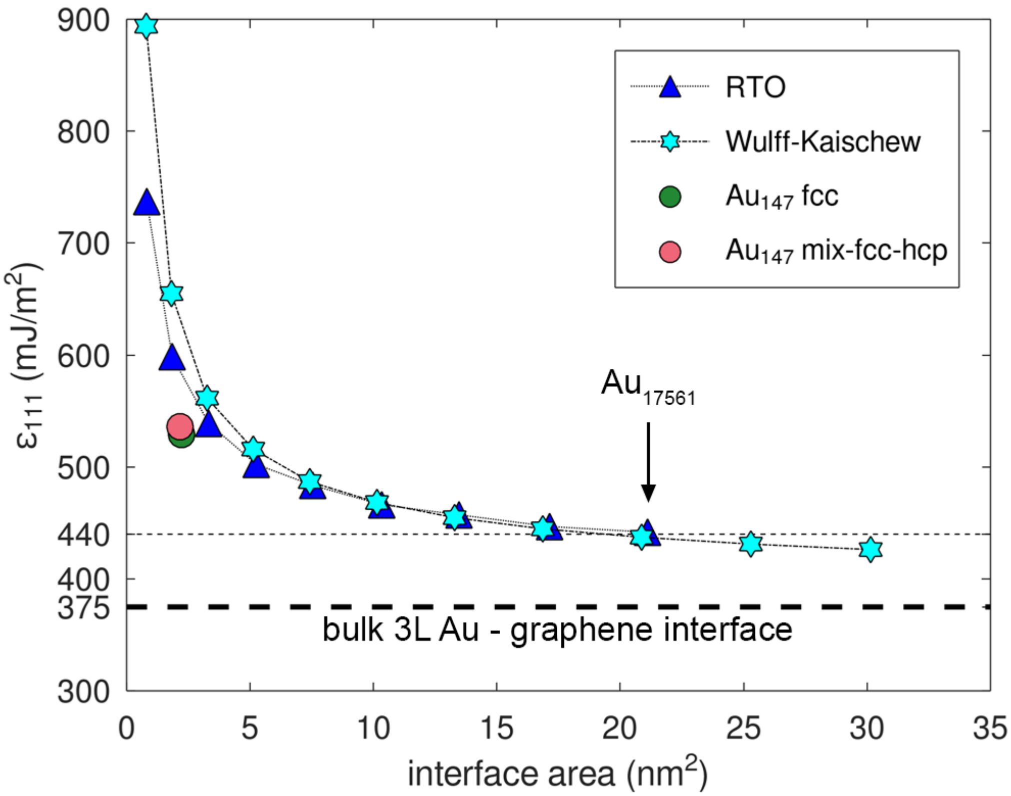

Figure S11: The adhesion energy per unit contact area (ε_{111}) as a function of the interface area of regular truncated octahedra (RTO) and of WK shapes. Thin horizontal line: the value of ε_{111} adopted in the determination of the WK shapes. Thick horizontal line: adhesion energy of bulk 3L Au - graphene interface [1].

Figure S11 reports the adhesion energy of regular truncated octahedra (RTO) Au clusters on graphene as a function of their contact area. The Au contact layers have a hexagonal shape with the same edge lengths. The contact area is calculated for this shape assuming all the Au-Au nearest neighbor distances equal to the mean Au-Au distance in the contact layer. Adhesion energy increases sharply as the interface area is decreased. As a result it is not straightforward to assign a single adhesion energy value for estimating the Wulff-Kaischew (WK) shapes. Based on the energy trend of RTO shapes, we adopt an estimation $\varepsilon_{111} = 440$ mJ$\cdot$ m^{-2} , which is close to the adhesion energy of Au_{17561}.
We then construct WK shapes based on this adhesion energy, and we report the adhesion energies of the WK structures too in Figure S11. The variation in adhesion energy of WK shapes follows approximately that of the RTO shapes.
For a few small clusters (Au_{49}, Au_{119}, and Au_{157}) we carry out Basin Hopping (BH) searches and confirmed the WK shape as the global minimum. These results give us confidence in the adopted value for the adhesion energy. In Figure S11, we also included the adhesion energy of the best fcc and best mix-fcc-hcp structures of Au_{147} along with the adhesion energy of bulk Au-graphene interface [1].

References

[1] W. Ouyang, O. Hod, R. Guerra, *J. Chem. Theory Comput.* **2021**, *17* 7215.
